# Circular RNA circTADA2A promotes osteosarcoma progression and metastasis by sponging miR-203a-3p and regulating CREB3 expression

**DOI:** 10.1186/s12943-019-1007-1

**Published:** 2019-04-02

**Authors:** Yizheng Wu, Ziang Xie, Junxin Chen, Jiaxin Chen, Weiyu Ni, Yan Ma, Kangmao Huang, Gangliang Wang, Jiying Wang, Jianjun Ma, Shuying Shen, Shunwu Fan

**Affiliations:** 1 Department of Orthopaedic Surgery, Sir Run Run Shaw Hospital, Zhejiang University School of Medicine, 3 East Qingchun Road, Hangzhou, 310016 Zhejiang Province China; 2 Key Laboratory of Musculoskeletal System Degeneration and Regeneration Translational Research of Zhejiang province, Hangzhou, 310016 Zhejiang Province China; 3 Biomedical Research Center and Key Laboratory of Biotherapy of Zhejiang Province, Sir Run Shaw Hospital, Zhejiang University School of Medicine, Hangzhou, 310016 China

**Keywords:** circTADA2A, Osteosarcoma, miR-203a-3p, CREB3, Metastasis

## Abstract

**Background:**

As a subclass of noncoding RNAs, circular RNAs (circRNAs) have been demonstrated to play a critical role in regulating gene expression in eukaryotes. Recent studies have revealed the pivotal functions of circRNAs in cancer progression. However, little is known about the role of circTADA2A, also named hsa_circ_0043278, in osteosarcoma (OS).

**Methods:**

CircTADA2A was selected from a previously reported circRNA microarray comparing OS cell lines and normal bone cells. QRT-PCR was used to detect the expression of circTADA2A in OS tissue and cell lines. Luciferase reporter, RNA immunoprecipitation (RIP), RNA pull-down and fluorescence in situ hybridization (FISH) assays were performed to confirm the binding of circTADA2A with miR-203a-3p. OS cells were stably transfected with lentiviruses, and Transwell migration, Matrigel invasion, colony formation, proliferation, apoptosis, Western blotting, and in vivo tumorigenesis and metastasis assays were employed to evaluate the roles of circTADA2A, miR-203a-3p and CREB3.

**Results:**

Our findings demonstrated that circTADA2A was highly expressed in both OS tissue and cell lines, and circTADA2A inhibition attenuated the migration, invasion and proliferation of OS cells in vitro as well as tumorigenesis and metastasis in vivo. A mechanistic study revealed that circTADA2A could readily sponge miR-203a-3p to upregulate the expression of CREB3, which was identified as a driver gene in OS. Furthermore, miR-203a-3p inhibition or CREB3 overexpression could reverse the circTADA2A silencing-induced impairment of malignant tumor behavior.

**Conclusions:**

CircTADA2A functions as a tumor promoter in OS to increase malignant tumor behavior through the miR-203a-3p/CREB3 axis, which could be a novel target for OS therapy.

**Electronic supplementary material:**

The online version of this article (10.1186/s12943-019-1007-1) contains supplementary material, which is available to authorized users.

## Background

Osteosarcoma (OS), a primary bone tumor arising from mesenchymal cells, has the highest fatality rate of all cancers among children and adolescents [1, 2]. Although advanced surgical techniques have been combined with multiple chemotherapies in clinical practice, the survival rate of OS remains unsatisfactory, and many patients suffer from disease recurrence due to existing or potential distant metastasis [3]. Patients with OS may benefit from additional therapies, such as small molecule-targeted drugs, but these strategies often lead to severe side effects and have failed in clinical trials [4, 5]. Therefore, new therapies for OS, especially therapies focusing on complex gene regulation axes or networks, are urgently needed.

As a special subclass of endogenous noncoding RNAs, circular RNAs (circRNAs) are RNAs made of closed covalent loops with a unique joining site between the 3′ and 5′ ends of the RNA formed by either canonical spliceosome-induced or noncanonical lariat-typed splicing, which is different from the formation process of linear RNA [6, 7]. Previous studies have exhibited the predominant evolutionary conservation and high abundance along with the structural stability of circRNAs across different species [8–10]. In the past several decades, multiple functions of circRNAs have been identified, including sponging microRNA, binding to RNA-binding proteins and protein translation functions [11–13]. In addition, circRNAs are involved in multiple cancer types and exert various influences on gene expression, apoptosis, and the cell cycle, among others [14]. Moreover, some circRNAs even have great potential as prognostic and diagnostic biomarkers for many diseases, especially cancer [15–17]. However, little is known about the roles and functions of circRNA in OS.

Cyclic AMP-responsive element-binding protein 3 (CREB3), also known as LZIP or LUMAN, is a member of the leucine zipper transcription factors. It exists ubiquitously in numerous cell types and can bind to the cAMP-response element and affect cell growth and proliferation [18]. Previous studies have revealed CREB3 to be a critical protein that interacts with host cell factor C1 encoded by herpes simplex virus (HSV), leading to the establishment of latency during HSV infection [19]. Furthermore, CREB3 plays a significant role in cervical cancer progression via the c-Jun-mmp9 axis and is involved in the malignant phenotype of prostate cancer [20, 21].

In this study, we identified circTADA2A from exons 5 and 6 of the Transcriptional Adaptor 2A (TADA2A) gene and characterized CREB3 as an oncogene in OS. We found that the expression of circTADA2A, but not that of TADA2A mRNA, was upregulated in both OS tissue and cell lines. Importantly, circTADA2A significantly enhanced the progression and metastasis of OS by sponging miR-203a-3p and targeting CREB3.

## Methods

### Ethical approval

All animal experiments were approved by the Ethics Committee of Sir Run Run Shaw Hospital and carried out under the guidelines of the Guide for the Care and Use of Laboratory Animals published by the National Institutes of Health.

### Clinical samples

OS tissue samples and chondroma tissue samples from 10 OS patients and 10 chondroma patients, respectively, were collected from the surgical specimen archives of Sir Run Run Shaw Hospital and the Second Affiliated Hospital of Zhejiang University School of Medicine, Zhejiang, China. All the procedures were approved by the Institutional Review Board of Sir Run Run Shaw Hospital and the Second Affiliated Hospital of Zhejiang University School of Medicine and conducted in accordance with the Declaration of Helsinki. All tissues were histologically characterized by pathologists in accordance with the criteria established by the World Health Organization and were stored in liquid nitrogen after surgery. Written informed consent was acquired from each patient before the beginning of this study.

### Cell culture and treatment

HEK-293 (ATCC: CRL-1573), human osteoblast hFOB1.19 (ATCC: CRL-11372) and human OS cells, including the HOS (ATCC: CRL-1543), 143B (ATCC: CRL-8303), MG-63 (ATCC: CRL-1427TM), U2OS (ATCC: HTB-96TM) and SJSA-1 (ATCC: CRL-2098) cell lines, were obtained from the American Type Culture Collection (ATCC, Manassas, VA, USA). All the cell lines, except for the hFOB1.19 cell line, were maintained in Dulbecco’s modified Eagle’s medium (DMEM) supplemented with 10% fetal bovine serum (Gibco, Grand Island, NY, USA), 100 U/mL penicillin, and 100 U/mL streptomycin (Invitrogen, Carlsbad, CA, USA). hFOB1.19 cells were maintained in Dulbecco’s modified Eagle’s medium/Nutrient Mixture F-12 (DMEM/F-12) supplemented with 10% fetal bovine serum (Gibco, Grand Island, NY, USA) and 0.3 mg/ml G418 (Invitrogen, Carlsbad, CA, USA). All the cells were cultured in an incubator at 37 °C with 5% CO2 and determined to be mycoplasma-free.

### SiRNAs, vector construction and stable transfection

SiRNAs were obtained from RiboBio (Guangzhou, China) and transfected into cells with Lipofectamine iMax (Invitrogen). Human lentivirus-sh-circTADA2A, lentivirus-miR-203a-3p sponge and lentivirus-pre-miR-203a-3p were purchased from GeneChem (Shanghai, China). The lentiviruses were ultracentrifuged, concentrated, validated and added to the cell culture medium. After infection, the cells were selected with puromycin (Gibco, Grand Island, NY, USA) for 1 week, and the surviving cells were continuously cultured as stable mass transfectants.

### RNA extraction, RNase R treatment, and qRT-PCR

Total RNA was extracted from certain cells and tissues using TRIzol (Invitrogen, Carlsbad, CA, USA) according to the kit instructions. For the RNase R treatment, 2 mg of total RNA was incubated for 15 min at 37 °C with or without 3 U/mg RNase R (Epicentre Technologies, Madison, WI, USA). For circRNA and mRNA analyses, a script RT reagent kit (TaKaRa) and SYBR Premix Ex Taq II (TaKaRa) were used, and the reactions were subsequently measured on a Roche LightCycler® 480II PCR instrument (Basel, Switzerland) in accordance with the manufacturer’s protocols. GAPDH was applied as an internal standard control. For miRNA analyses, the samples were treated with DNase I to eliminate genomic DNA, and cDNA was synthesized by using the Mir-X miR First-Strand Synthesis Kit (TaKaRa). SYBR Premix Ex Taq II (TaKaRa) was used for qRT-PCR. U6 was used as an internal standard control. The relative RNA expression levels were analyzed using the 2^-△△Ct^ method. Three experiments were performed with three replicates each.

### Nucleic acid electrophoresis

The cDNA and gDNA PCR products were investigated using 2% agarose gel electrophoresis with TAE running buffer. DNA was separated by electrophoresis at 120 V for 30 min. The DNA marker used was Super DNA Marker (CWBIO, Beijing, Cat. CW2583M). The bands were examined by UV irradiation.

### Fluorescence in situ hybridization (FISH)

Alexa Fluor 555-labeled circTADA2A probes and Alexa Fluor 488-labeled miR-203a-3p probes were designed and synthesized by RiboBio (Guangzhou, China). The probe signals were determined with the Fluorescent in Situ Hybridization Kit (RiboBio, Guangzhou, China) according to the manufacturer’s guidelines. Images were acquired using a fluorescence microscope (Eclipse E600; Nikon Corporation, Tokyo, Japan). The expression level of miR-203a-3p in tissues was evaluated by FISH using a miR-203a-3p-specific probe on tissue arrays containing 56 samples (Alenabio, China). Quantitative scanning method to assess the expression of miR-203a-3p was Aperio ImageScope V11 from Leica Company [22, 23], and the positivity value × 100 represents the expression level of miR-203a-3p.

### Predicted miRNA targets of circTADA2A

We predicted the miRNA binding sites of circTADA2A using the bioinformatics databases miRanda (http://www.microrna.org), TargetScan (http://www.targetscan.org/), RNAhybrid (https://bibiserv.cebitec.uni-bielefeld.de/rnahybrid/) and circBank (http://www.circbank.cn). Filtering restrictions were as follows: (i) Total score ≥ 140 and Total energy < 4 kcal/mol; (ii) Number of estimated binding sites > 1; and (iii) Minimum free energy (MFE) ≤0 kcal/mol.

### Pull-down assay with a biotinylated circTADA2A probe

A pull-down assay was performed as indicated. Briefly, 1 × 10^7^ HOS and 143B cells were collected, lysed, and sonicated. Probe-coated beads were generated by coincubating the circTADA2A probe with C-1 magnetic beads (Life Technologies) at 25 °C for 2 h. The cell lysates were incubated with the circTADA2A probe or oligo probe at 4 °C overnight. After washing with wash buffer, the RNA complexes bound to the beads were eluted and extracted with the RNeasy Mini Kit (QIAGEN) for RT-PCR or qRT-PCR. The biotinylated circTADA2A probe was designed and synthesized by RiboBio (Guangzhou, China).

### RNA immunoprecipitation (RIP)

RIP experiments were performed using the Magna RIP RNA-Binding Protein Immunoprecipitation Kit (Millipore, Billerica, MA, USA). HEK-293 cells were transfected with the Ago2 plasmid or vector. Then, 1 × 10^7^ cells were pelleted and resuspended in 100 μl of RIP Lysis Buffer combined with a protease inhibitor cocktail and RNase inhibitors. The cell lysates (200 μl) were incubated with 5 μg of antibody against Ago2 (Millipore) or rabbit IgG-coated beads and rotated at 4 °C overnight. After treating the lysates with proteinase K buffer, immunoprecipitated RNA was extracted by using the RNeasy MinElute Cleanup Kit (Qiagen) and reverse transcribed using Prime-Script RT Master Mix (TaKaRa). The abundance of circTADA2A was detected by qRT-PCR.

### Luciferase reporter assay

The reporter plasmids (pGL3-Firefly_Luciferase-Renilla_Luciferase containing the circTADA2A sequence or a mutant sequence and pGL3-Firefly_Luciferase-Renilla_Luciferase containing the CREB3 sequence or a mutant sequence) were designed by Shanghai GeneChem Co. HEK-293 cells were cotransfected with the reporter plasmid and miR-203a-3p mimic or negative control mimic (mimic N.C.) (RiboBio, Guangzhou, China) and incubated for 24 h. For comparisons, firefly luciferase activity was normalized to Renilla luciferase activity. The effect of a miRNA on the luciferase reporter with the circTADA2A 3′-UTR, CREB3 3′-UTR or the corresponding mutant was calculated by comparing the reporter with the control.

### Transwell migration and Matrigel invasion assays

The Transwell migration and Matrigel invasion assays were performed with a Transwell chamber (for the migration assay) or a Transwell chamber precoated with Matrigel (for the invasion assay) according to the manufacturer’s protocols (BD Biosciences, Bedford, MA, USA). Briefly, 200 μL of serum-free medium containing 5 × 10^4^ treated cells for the migration assay or 1 × 10^5^ treated cells for the invasion assay was added to the upper chambers, and 600 μL of complete medium was added to the lower chambers, and then the cells were incubated for 24 h. Representative images were obtained under an inverted light microscope (Zeiss, Primovert). Migrated and invaded cells were quantified by counting the number of migrated or invaded cells in at least three random fields of view.

### Colony formation assay

Transfected cells were seeded at a density of 600 cells/well into a 12-well plate and then cultured for 8 days. Then, the cells were washed with phosphate-buffered saline (PBS), fixed with 4% paraformaldehyde for 20 min and stained with a 0.5% crystal violet solution for another 20 min. The colonies were counted and imaged under a microscope. The experiments were replicated at least three times.

### Wound-healing assay

OS cells were cultured in six-well plates, and cell monolayer was subsequently scratched with a 200 μl pipette tip. Representative images of cell migration were captured by photographing 10 high-power fields at 0 and 48 h after injury. Remodeling was measured as the diminishing distance across the induced injury area normalized to the 0 h control and expressed as a relative migration rate. The experiments were repeated at least three times with three replicates each.

### Soft agar colony formation assay

Stable OS cells were isolated and seeded in semisolid agar medium (0.5% agarose/PBS culture medium with a 0.6% agarose/PBS bottom layer in a 6-well plate) at a density of 25,000 cells/well. After 0, 7, or 14 days of incubation, representative images of the cell colonies were obtained under an inverted light microscope (Zeiss, Primovert).

### Apoptosis analysis

Apoptosis was determined by using an Annexin V-FITC/propidium iodide (PI) kit (BD Biosciences, San Diego, CA, USA). In brief, cells were trypsinized, washed with ice-cold PBS, and stained with Annexin V-FITC/PI. After a 15 min incubation, the cells were analyzed using a flow cytometer (BD FACSCANTO II, BD Biosciences, San Jose, CA, USA) and FlowJo software.

### Zymography assay

Conditional media from different stably transfected cells were collected, standardized and mixed with zymogram sample buffer (BioRad, California, USA) without heating. Samples were loaded and separated by 10% SDS-PAGE gels containing 1 mg/mL gelatin under nonreducing conditions. After separation by electrophoresis, the gel was washed with renaturation buffer (BioRad, California, USA) and incubated with a buffer containing 50 mM Tris-HCl, pH 8.0, 5 mM CaCl2, and 0.02% NaN3 at 37 °C. Then, the gel was stained with Coomassie blue R-250 for 60 min and destained with destaining buffer. The image was taken with the GeneSys (version 1.2.0.0) software.

### Western blotting analysis and antibodies

Cells were lysed in RIPA Lysis Buffer (MA0151, Dalian Meilun Biotechnology Co., Ltd., China) containing a protease inhibitor cocktails (FD1001, Fudebio, Hangzhou, China) on ice. Equal amounts of total protein from different samples were separated by SDS-PAGE gels at 100 V for 1.5 h and transferred onto 0.22 μm polyvinylidene difluoride (PVDF) membranes (Amersham Bioscience, Piscataway, NJ) at 280 mA for 1.5 h. Then, the membranes were blocked with 5% skim milk powder in TBST for 1 h at room temperature and treated with specific primary antibodies overnight at 4 °C. The next day, the membranes were washed with TBST and incubated with an HRP-conjugated secondary antibody (FDM007 and FDR007, Fudebio, Hangzhou, China). Each band was detected using an enhanced chemiluminescence kit (FD8030, Fudebio, Hangzhou, China). Anti-GAPDH, anti-CREB3, anti-Bcl-2, anti-mmp2, anti-Vimentin and anti-E-cadherin antibodies were purchased from Abcam (Cambridge, UK). The anti-c-Jun, anti-cleaved-caspase 3 antibodies were purchased from Cell Signaling Technology (Boston, MA, USA); anti-mmp9 antibody was purchased from ABclonal (Boston, MA, USA); and anti-N-cadherin antibody was purchased from Proteintech (Chicago, USA). Primary Antibody Dilution Buffer was purchased from Dalian Meilun Biotechnology Co., Ltd. (MB9881, Dalian, China).

### Subcutaneous and orthotopic xenograft tumor models

Nude mice (male, 4 weeks old) were used for the in vivo tumor models. A total of 5 × 10^6^ 143B stable cells were injected subcutaneously or into the cavity of the tibia (cells were labeled with a luminescent dye and GFP). Tumor volume was calculated using the following formula: volume (mm^3^) = ab^2^/2. Thirty days after injection, the animals were sacrificed, and the tumors were harvested and fixed in 4% paraformaldehyde. Tumor weight is shown as the mean ± SEM of each group.

### Tail vein metastasis model and CTC (circulating tumor cell) detection

Luminescence and GFP-labeled 143B cells were stably transfected with N.C. or sh-circTADA2A or cotransfected with sh-circTADA2A and the miR-203a-3p sponge. The three different stable 143B cell lines (equivalent volume and density) were suspended in sterile PBS and injected into the tail vein of each nude mouse (male, 4 weeks old). After 4 weeks, lung metastasis was evaluated via an in vivo bioluminescence imaging system. Blood from the mice were collected, and the red blood cells were lysed using red blood cell lysis buffer. An equal volume of each sample was analyzed via flow cytometry. The percentage of GFP-positive cells reflects the CTCs in the circulation. Representative images of the samples containing CTCs or CTC cluster were obtained from fluorescence microscope.

### Other in vitro experiments

The other in vitro experiments performed have been previously described and included CCK-8 assay, H&E staining, TUNEL assay, immunohistochemistry (IHC) and immunofluorescence microscopy [24, 25].

### Statistical analyses

Statistical analyses were performed with SPSS 20 software (Abbott Laboratories, Chicago, IL, USA). Data were analyzed with unpaired Student’s t-test unless indicated otherwise. The results are presented as the mean ± SEM. *P* values less than 0.01 were considered statistically significant.

## Results

### CircTADA2A is relatively highly expressed in OS tissues and cell lines and is predominantly localized in the cytoplasm

**Fig. 1:**
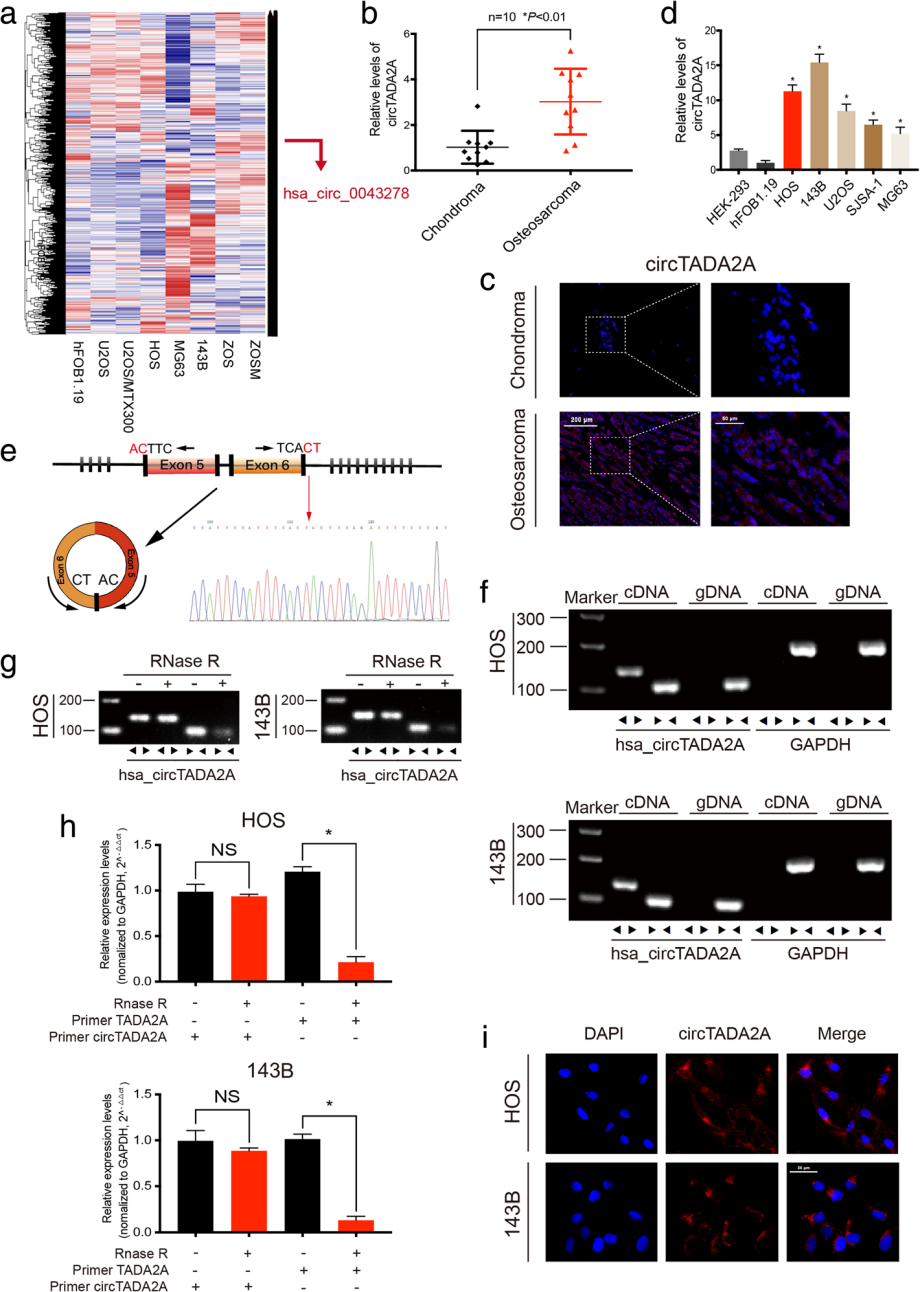
The validation and expression of circTADA2A in osteosarcoma tissues and cells. **a** CircRNA microarray based on osteosarcoma cell lines and hFOB1.19 in GSE96964. **b** The expression of circTADA2A was detected by qRT-PCR in 10 osteosarcoma and chondroma tissues (*n* = 10) (*P < 0.01, Student’s *t*-test). **c** Representative FISH images demonstrating circTADA2A expression detected by a junction probe in chondroma and osteosarcoma tissues; scale bars, 200 μm and 50 μm (FISH, fluorescence in situ hybridization). **d** CircTADA2A expression was detected by qRT-PCR in various human osteosarcoma cell lines (HOS, 143B, U2OS, SJSA-1 and MG63) and normal cells (osteoblast hFOB1.19 and HEK-293); circTADA2A mRNA levels were higher in OS cells than in hFOB1.19 and HEK-293 cells. **e** Schematic illustration demonstrates the formation of circTADA2A via the circularization of exons 5 and 6 in TADA2A (black arrow). The presence of circTADA2A was validated by RT-PCR, followed by Sanger sequencing. The head-to-tail splicing site of circTADA2A is indicated by the red arrow. **f** RT-PCR validated the existence of circTADA2A in HOS and 143B cell lines. CircTADA2A was amplified by divergent primers in cDNA but not gDNA. GAPDH was used as a negative control. **g & h** The expression of circTADA2A and TADA2A mRNA in both HOS and 143B cell lines was detected by RT-PCR or qRT-PCR in the presence or absence of RNase R. **i** FISH showed that circTADA2A was predominantly localized in the cytoplasm. Nuclei were stained with DAPI, and circTADA2A probes were labeled with Alexa Fluor 555; scale bars, 50 μm. Data are from three independent experiments (mean ± SEM) (**d** and **h**) or are representative of three independent experiments with similar results (**c**, **f**, **g** and **i**) (*P < 0.01 vs control or as indicated by Student’s *t-*test)

A microarray expression profile comparing circRNAs in OS cell lines with those in hFOB1.19 cells has been described previously (GSE96964) [26]. We found that the expression level of hsa_circ_0043278, also named as circTADA2A, was significantly increased in various OS cell lines compared with hFOB1.19 cells, a normal osteoblast cell line (Fig. 1a). To investigate the correlation between circTADA2A expression and OS, we collected 10 pairs of chondroma and OS tissue samples and used qRT-PCR to detect circTADA2A expression. Figure 1b demonstrates the relative abundance of circTADA2A in OS tissue compared with chondroma tissue, and this difference in expression was further verified by FISH (Fig. 1c). Consistent with the results of the clinical samples, the expression circTADA2A was obviously higher in multiple OS cell lines (HOS, 143B, U2OS, SJSA-1, and MG63) than in the hFOB1.19 cell line and HEK-293 cells. Among the OS cell lines, HOS and 143B cells exhibited the highest levels of circTADA2A (Fig. 1d).

By comparing TADA2A mRNA sequences with the expected sequences of circTADA2A acquired from circBase, we determined that circTADA2A was looped and comprised exons 5 and 6 of its parental gene. We further confirmed the head-to-tail splicing via Sanger sequencing (Fig. 1e). However, head-to-tail splicing could be the result of not only trans-splicing but also genomic rearrangements. To distinguish between these two possibilities, convergent primers for TADA2A mRNA and special divergent primers to amplify circTADA2A were designed. CDNA and gDNA were extracted separately from HOS and 143B cells and subjected to nucleic acid electrophoresis detection, and results indicated that circTADA2A could be detected only in cDNA, as no products were detected in the extracted gDNA (Fig. 1f). Stability is considered one of the crucial characteristics of circRNAs [6–8]. To confirm the stability of circTADA2A, RNase R was employed in the experiments. As shown in Fig. 1g and h, the levels of the linear forms of TADA2A decreased sharply under the RNase R treatment, but RNase R failed to digest circTADA2A. Moreover, RNA FISH revealed that circTADA2A was mainly localized in the cytoplasm (Fig. 1i).

### Silencing circTADA2A inhibits the migration, invasion and proliferation of OS cells

**Fig. 2:**
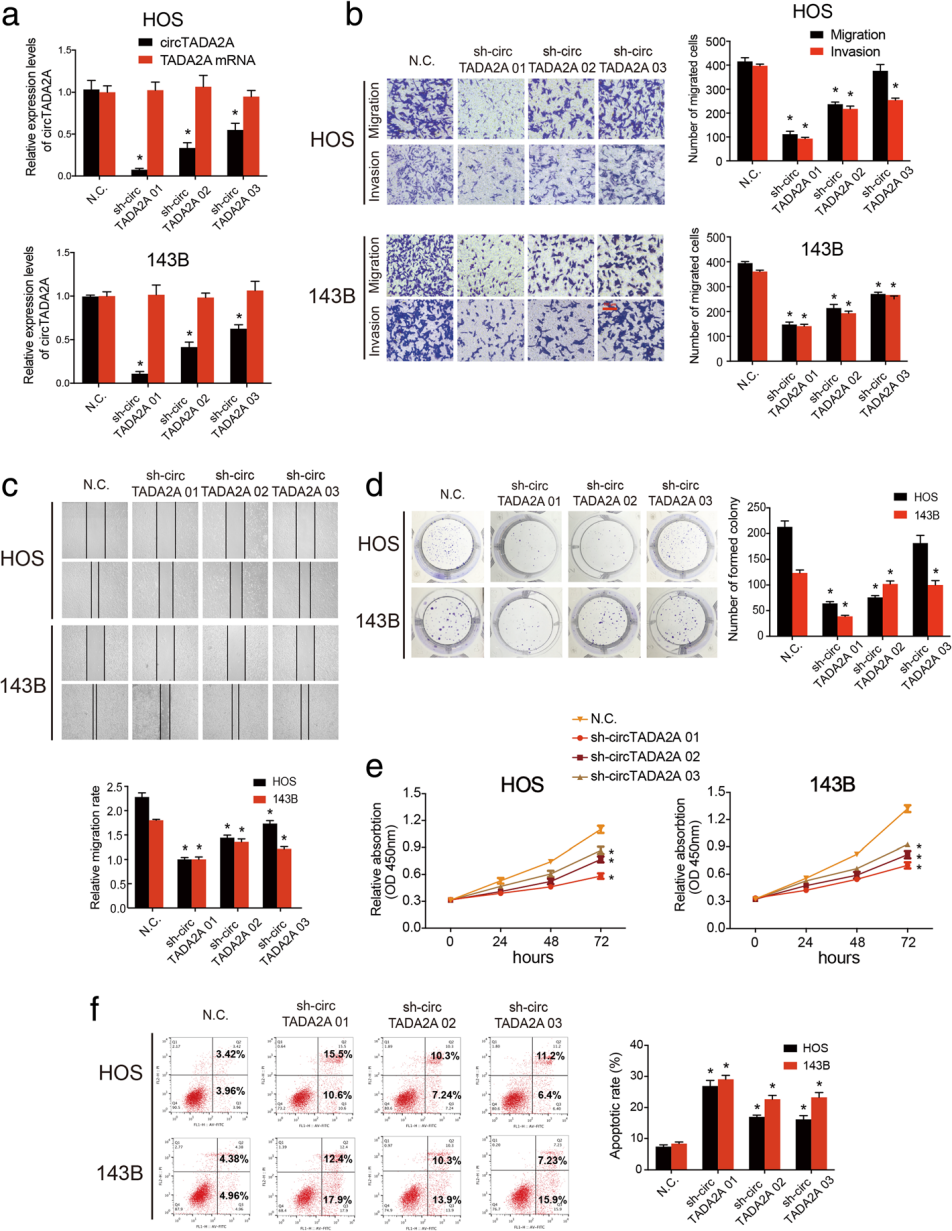
CircTADA2A affects the migration, invasion, proliferation abilities of osteosarcoma. **a** The expression of circTADA2A and TADA2A mRNA in HOS and 143B cells after stable transfection of circTADA2A sh-circTADA2A or N.C. (vector plasmids) were detected by qRT-PCR. **b** Transwell migration and Matrigel invasion assays were used to measure the migration and invasion ability of stable HOS and 143B cells. Representative images are shown; scale bars, 100 μm. **c** CircTADA2A knockdown suppressed cell migration capacity, as determined by the wound-healing assay. **d** Colony formation assay was used to determine the ability of proliferation in the stably transfected HOS and 143B cells. **e** ShRNA-mediated circTADA2A silence suppressed OS cell growth, as determined by the CCK-8 assay. **f** HOS and 143B cells were transfected with sh-circTADA2A, followed by Annexin V-FITC/PI staining. The percentage of apoptotic cells is shown. Data are from three independent experiments (mean ± SEM) (**a-f)** (*P < 0.01 vs control or as indicated by Student’s *t*-test)

CircTADA2A small hairpin RNAs (shRNAs), which could stably knock down the expression of circTADA2A in most cells, were generated to explore the function of circTADA2A in OS cells. We designed 3 sh-circTADA2A products that specifically targeted the junction sites of this circRNA, transfected them into HOS and 143B cells, and then assessed the transfected cells by qRT-PCR. As shown in Fig. 2a, circTADA2A expression was significantly silenced by shRNA, while the expression of circTADA2A mRNA did not change. Among the shRNAs, sh-circTADA2A 01 had the best knockdown efficiency. The migration and invasion abilities of the OS cells were prominently decreased by the circTADA2A shRNAs, and these effects were confirmed by the wound-healing assay (Fig. 2b and c). Then, alterations in proliferative capacity were further evaluated. The results of the colony formation assay demonstrated that the knockdown of circTADA2A expression significantly suppressed the colony-forming ability of OS cells (Fig. 2d). In addition, cell viability was further evaluated by a CCK-8 assay, which indicated that circTADA2A had a critical influence on maintaining a high proliferation rate (Fig. 2e). Moreover, we also performed an apoptosis assay, suggesting that apoptosis in OS cells could be triggered by circTADA2A silencing (Fig. 2f). In the experiments described above, sh-circTADA2A 01 was found to have the most powerful effect, which is consistent with the knockdown efficiency observed by qRT-PCR. Thus, sh-circTADA2A 01 was chosen for further study. Taken together, these findings reveal the momentous role of circTADA2A in the motility and proliferation of OS cells in vitro*.*

### CircTADA2A functions as an efficient miR-203a-3p sponge in OS

**Fig. 3:**
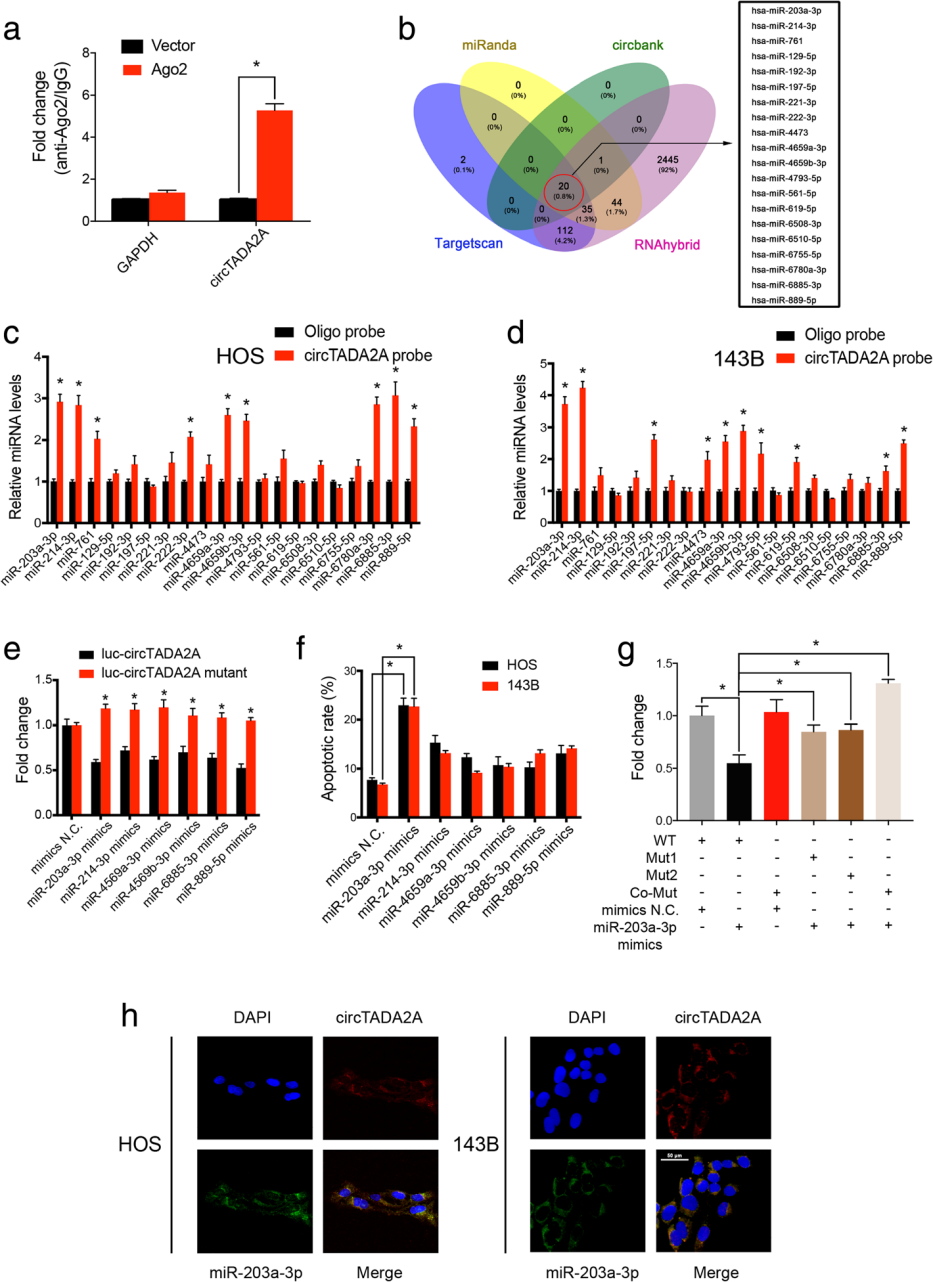
CircTADA2A acts as a sponge for miR-203a-3p in osteosarcoma cells. **a** RIP assay for circTADA2A levels in HEK-293 cells transfected with Ago2 (RIP, AGO2 RNA immunoprecipitation). **b** Schematic illustration exhibiting overlapping of the target miRNAs of circTADA2A predicted by miRanda, circbank, TargetScan, and RNAhybrid. **c & d** The relative levels of 20 miRNA candidates in the HOS and 143B lysates were examined by qRT-PCR. Multiple miRNAs can be pulled down by circTADA2A. **e** Luciferase activities of luc-circTADA2A or luc-circTADA2A-mutant in HEK-293 cells cotransfected with 6 selected miRNA mimics or mimics N.C. were determined by a luciferase reporter assay. **f** Apoptosis assay with Annexin V-FITC/PI staining was performed to analyze the apoptotic rates of HOS and 143B cells transfected with 6 selected miRNA mimics or mimics N.C. **g** Luciferase reporter assay was performed to detect the luciferase activities of HEK-293 cells cotransfected with a luciferase reporter construct containing wild-type (or mutant) circTADA2A and miR-203a-3p mimics (or mimics N.C.). **h** Colocalization between miR-203a-3p and circTADA2A was observed via FISH in both HOS and 143B cells. CircTADA2A probes were labeled with Alexa Fluor 555. MiR-203a-3p probes were labeled with Alexa Fluor 488. Nuclei were stained with DAPI; scale bar, 50 μm. Data are from three independent experiments (mean ± SEM) (**a and c-g**) or are representative of three independent experiments with similar results (**h**) (*P < 0.01 vs control or as indicated by Student’s *t-*test)

Previous studies have reported that circRNAs can function as miRNA sponges and subsequently abrogate the function of the corresponding miRNA [11, 27–30]. Given that circTADA2A predominantly localized in the cytoplasm and exhibited marked stability, we next explored whether circTADA2A enhanced the biological behavior of OS by sponging miRNAs. HEK-293 cells were transfected with the AGO2 plasmid or vector and then used in a RIP assay with antibodies targeting AGO2. By qRT-PCR, we found that the endogenous circTADA2A pulled down by anti-AGO2 antibodies was predominantly enriched in the AGO2 overexpression group compared with the control group, suggesting that circTADA2A interacts and binds with miRNAs through AGO2 protein (Fig. 3a). Four databases (miRanda, circBank, TargetScan and RNAhybrid) were used to predict the potential target miRNAs, among which 20 miRNAs were selected from the overlap between the databases (Fig. 3b). Then, we performed a pull-down assay with a biotinylated circTADA2A probe and selected 6 possible miRNAs with significantly enhanced fold-changes for circTADA2A capture in both HOS and 143B cells (Fig. 3c and d). As expected, the binding of 6 miRNAs was further confirmed by the results of the luciferase assay (Fig. 3e).

We next examined the function of these miRNAs in OS cells via an apoptosis assay. As shown in Fig. 3f and Additional file 1: Figure S1a, the overexpression of miR-203a-3p resulted in the highest apoptosis rates in both the HOS and 143B cell lines after 48 h, while fewer apoptotic cells were detected with the overexpression of the other miRNAs. Bioinformatic analysis using the CircInteractome database revealed that circTADA2A shares 2 miRNA response elements with miR-203a-3p. We subsequently mutated these response elements and cloned them into a luciferase reporter containing the 3′-untranslated region (3′-UTR) of circTADA2A (Additional file 1: Figure S1b). MiR-203a-3p was transfected into HEK-293 cells, and we found that the luciferase activities of the Mut1 and Mut2 reporters were significantly higher than those of the wild-type (WT) reporter. By comparison, the reporter containing the comutant with the 2 mutated binding sites had a much stronger luciferase activity than any of the other reporters (Fig. 3g), indicating that miR-203a-3p can directly bind to circTADA2A. Furthermore, RNA FISH revealed that miR-203a-3p and circTADA2A colocalized in the cytoplasm of the OS cells (Fig. 3h), indicating that miR-203a-3p can be sponged by circTADA2A.

### MiR-203a-3p is downregulated in OS cells and tissues and inhibits the malignant behavior of OS

**Fig. 4:**
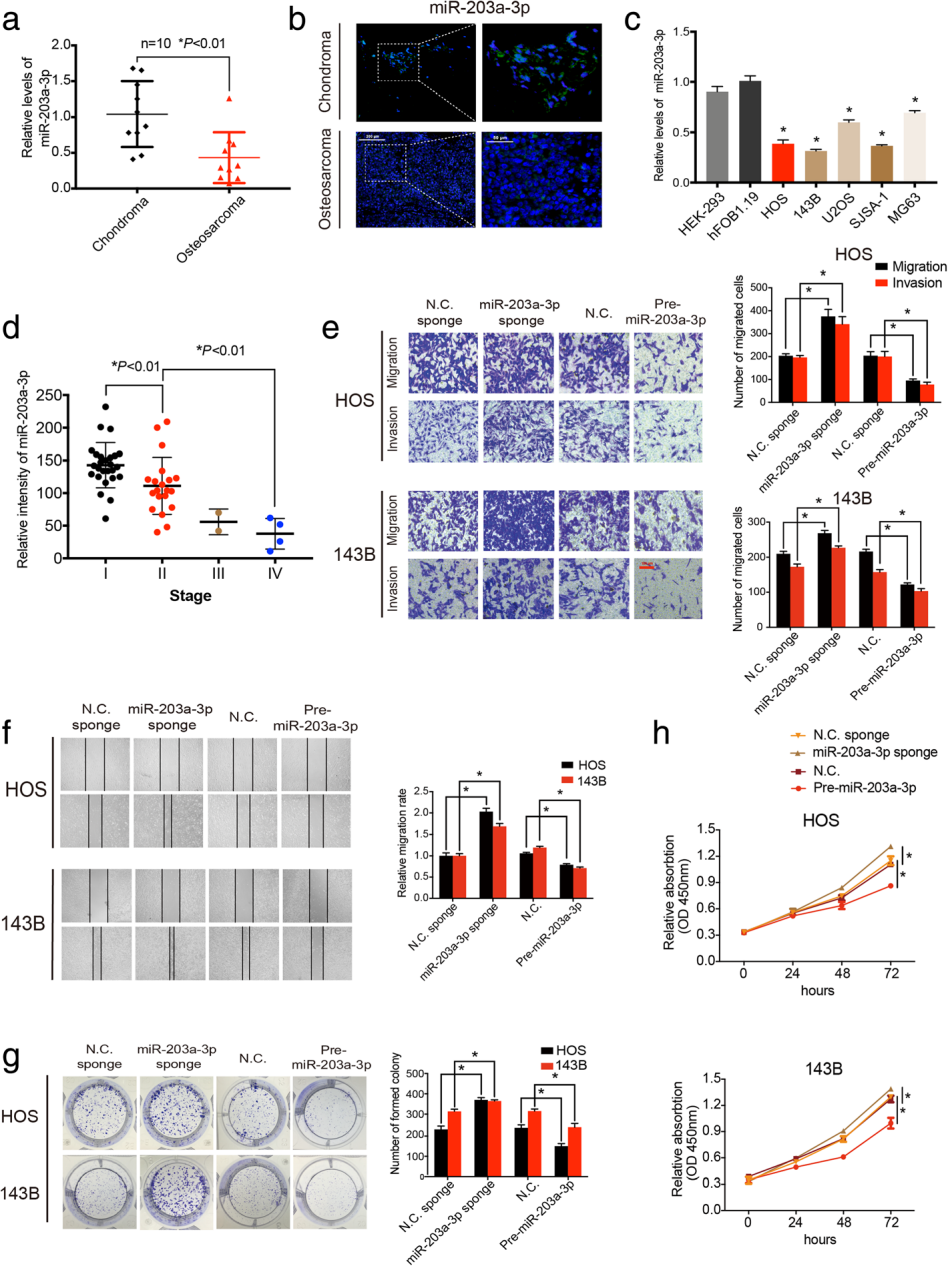
MiR-203a-3p regulates the migration, invasion and proliferation of OS cells. **a** The expression of miR-203a-3p was detected by qRT-PCR assay in 10 osteosarcoma and chondroma tissues (*n* = 10) (*P < 0.01, Student’s *t*-test). **b** FISH showed the miR-203a-3p expression level was lower in osteosarcoma tissue than in chondroma tissue; scale bars, 200 μm and 50 μm. **c** MiR-203a-3p expression was detected by qRT-PCR in various human osteosarcoma cell lines (HOS, 143B, U2OS, SJSA-1 and MG63) and normal cells (osteoblast hFOB1.19 and HEK-293). **d** The expression of miR-203a-3p in OS at different clinicopathological stages was analyzed by FISH on an OS tissue chip (56 cases). Intensity of miR-203a-3p expression was calculated from the OS tissue chip. **e** The effect of miR-203a-3p on cell migration and invasion was evaluated by Transwell migration and Matrigel invasion assay. Representative images were shown. Scale bars, 100 μm. **f** Representative images of the wound-healing assay exhibiting changes in the migration capacity of stable OS cells. **g** Proliferation ability of stably transfected OS cells was evaluated by colony formation assay. Representative images are shown. **h** pre-miR-203a-3p (or miR-203a-3p sponge)-mediated miR-203a-3p overexpression (or knockdown) influenced the OS cell viability, as determined by a CCK-8 assay. Data are from three independent experiments (mean ± SEM) (**c, e-h**) or are representative of three independent experiments with similar results (**b**) (*P < 0.01 vs control or as indicated by Student’s t-test)

The expression of miR-203a-3p in both the OS tissue (Fig. 4a) and cell lines (Fig. 4c) was detected using qRT-PCR. The results revealed that miR-203a-3p expression was significantly decreased in the OS tissue samples compared with the chondroma tissue samples, and this pattern was further confirmed by RNA FISH (Fig. 4b). Additionally, miR-203a-3p exhibited lower expression level in the OS cell lines than in the hFOB1.19 and HEK-293 cells. As miR-203a-3p expression is significantly reduced in OS tissues, we next analyzed whether miR-203a-3p decrease is associated with the clinicopathological stages of OS patients. A cohort of 52 OS patients with survival data was included, and miR-203a-3p expression was determined via FISH analysis on tissue array slides. As shown in Fig. 4d, miR-203a-3p expression was negatively correlated with OS clinicopathological stages. MiR-203a-3p expression in OS samples at stage II was significantly downregulated compared with stage I and was much higher than stage IV. Considering only 2 samples in stage III were collected, no significant difference between stage II and stage III as well as stage III and stage IV were found; however, an obvious decreasing tendency in miR-203a-3p from stage I to stage IV could be observed.

Then, we found that the mRNA expression of miR-203a-3p was significantly upregulated by circTADA2A knockdown (Additional file 2: Figures S3a and S3b). Considering that circTADA2A is able to interact with miR-203a-3p, we then evaluated the role of miR-203a-3p in OS by constructing HOS and 143B cells with stable overexpression of miR-203a-3p via pre-miR-203a-3p or with stable knockdown of miR-203a-3p via miR-203a-3p sponging. Transfection efficiency was verified by qRT-PCR (Additional file 3: Figures S2a and S2b). The results of the Transwell migration and Matrigel invasion assays along with wound-healing assays revealed that miR-203a-3p predominantly abrogated the migratory and invasive abilities of the OS cells (Fig. 4e and f). Epithelial-mesenchymal transition (EMT) is a genetic program aberrantly activated in various cancers that is characterized by the loss of cell-cell adhesion protein E-cadherin and the acquisition of mesenchymal biomarkers such as N-cadherin, Vimentin and mmp2 [31–34]. Western blotting results demonstrated that miR-203a-3p could upregulate the protein level of E-cadherin and downregulate the expression of N-cadherin, Vimentin and mmp2, indicating that EMT process was attenuated with miR-203a-3p overexpression (Fig. 6k and Additional file 4: Figures S6d and S6f). We also investigated mmp2 and mmp9 activity via zymography assay (Additional file 3: Figure S2c). The results indicated that both mmp2 and mmp9 activity were enhanced by the miR-203a-3p sponge, while the overexpression of miR-203a-3p could attenuate this activity, as shown both in pro and active form of mmp2 and mmp9. In addition to metastatic ability, proliferation ability was examined, and we found that the forced expression of miR-203a-3p obviously reduced cell proliferation, while the downregulation of miR-203a-3p promoted cell viability in the colony formation assay and CCK-8 assay (Fig. 4g and h). Overall, these data indicate that miR-203a-3p suppresses the migration, invasion and proliferation of OS cells in vitro.

### Silencing miR-203a-3p reverses the sh-circTADA2A-induced antitumor effects in OS cells

**Fig. 5:**
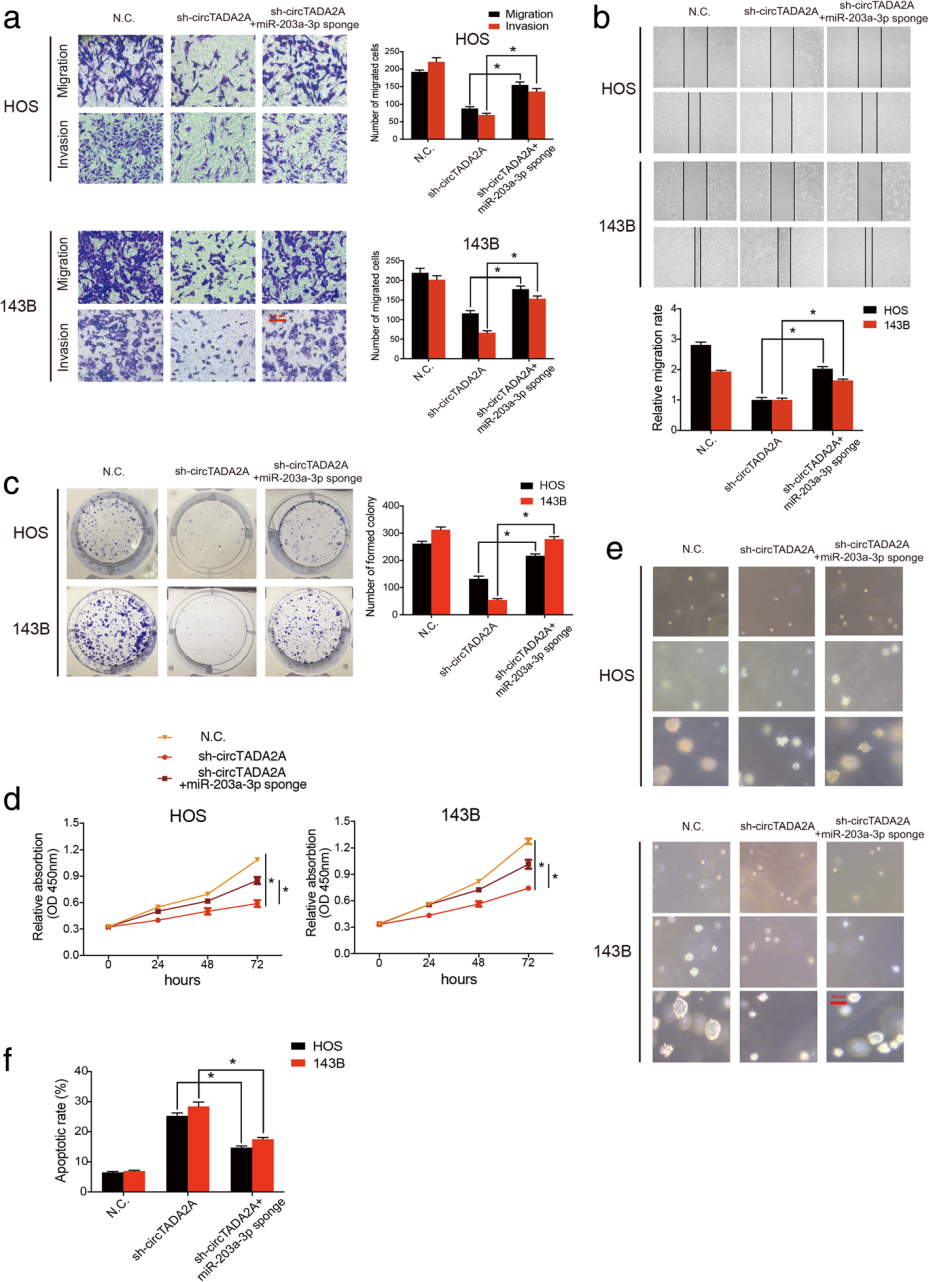
Knockdown of miR-203a-3p reverses sh-circTADA2A-induced the attenuation of cell migration, invasion and proliferation in OS cells. **a** HOS and 143B cells were stably transfected with N.C. or sh-circTADA2A or cotransfected with sh-circTADA2A and miR-203a-3p sponge. The migration and invasion ability was evaluated by Transwell migration and Matrigel invasion assay. Scale bars, 100 μm. **b** Wound-healing assay demonstrated the reversion of migration ability by miR-203a-3p sponge. Representative images are shown. **c** Colony formation assay demonstrated the effect of sh-circTADA2A and the miR-203a-3p sponge on the colony-forming ability of OS cells. **d** Proliferation of OS cells transfected with control N.C. and sh-circTADA2A with or without the miR-203a-3p sponge was evaluated by a CCK-8 assay. **e** Representative images of soft agar colony formation assay demonstrated the proliferation ability of stable OS cells at 0, 7, and 14 days. Scale bars, 100 μm. **f** The effects of circTADA2A knockdown and miR-203a-3p sponge rescue on circTADA2A silence were evaluated by apoptosis assay. Apoptotic rates were determined by Annexin V-FITC/PI staining. Data are from three independent experiments (mean ± SEM) (**a-d and f**) or are representative of three independent experiments with similar results (**e**) (*P < 0.01 vs control or as indicated by Student’s t-test)

**Fig. 6:**
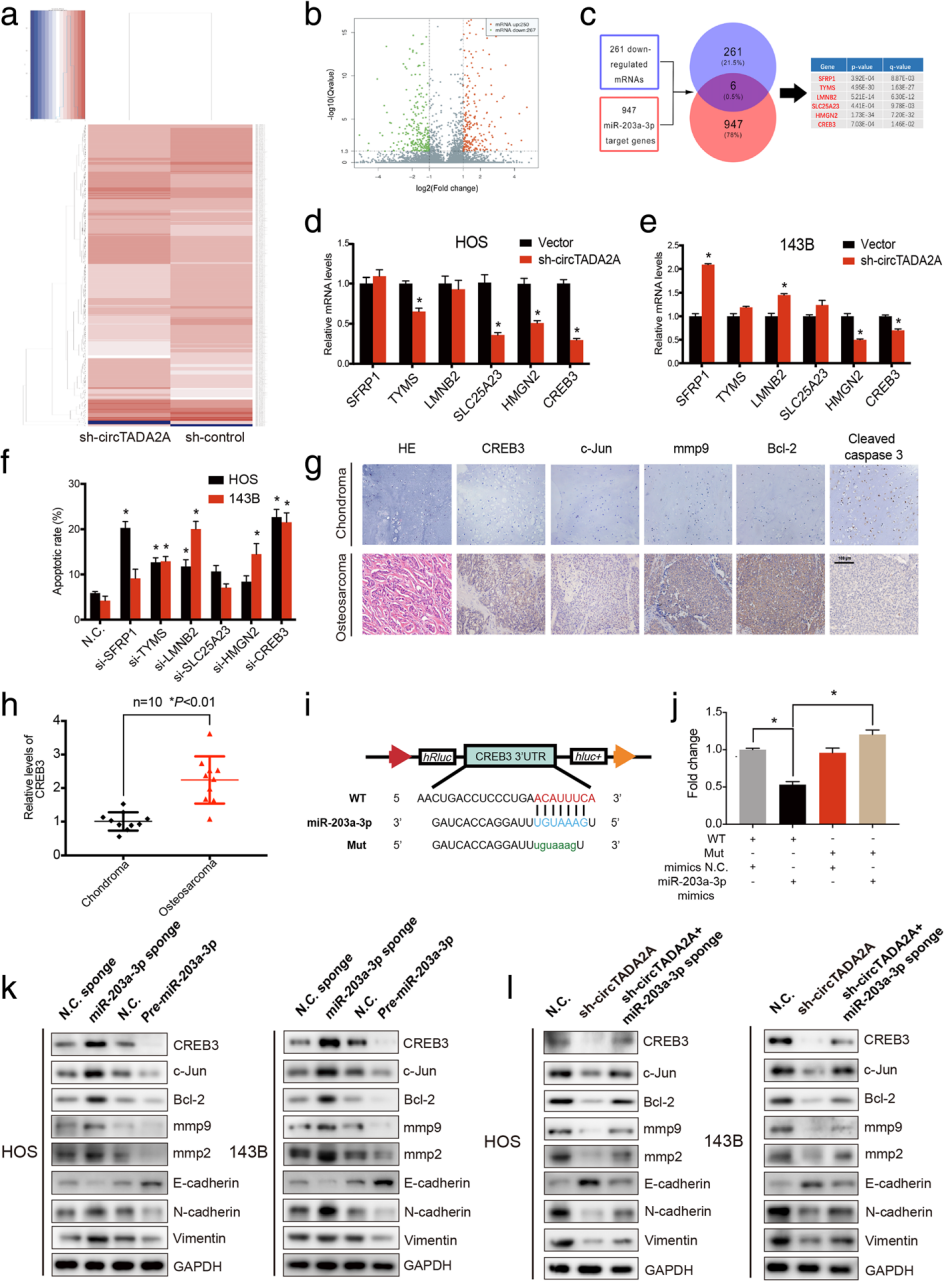
CREB3 is a direct target of miR-203a-3p. **a** Clustered heatmap of significantly differentially expressed mRNAs in HOS transfected with sh-control and with sh-circTADA2A. Each sample was mixed with three replicates. **b** Significantly overexpressed genes are represented by red dots, and significant downregulated genes are represented by green dots in the volcano plot. **c** Schematic flowchart shows the overlapping of the downregulated mRNAs collection and target gene analyses of miR-203a-3p from TargetScan database. **d & e** The expression levels of 6 selected genes in HOS and 143B cells transfected with N.C. or sh-circTADA2A were determined by qRT-PCR. The histograms show relative mRNA levels. **f** Both HOS and 143B cells were transfected with siRNA of 6 selected genes. Apoptotic rates after 48 h were evaluated by Annexin V-FITC/PI staining. **g** H&E staining and immunohistochemistry (IHC) results of chondroma and OS tissues. CREB3, c-Jun, mmp9 and Bcl-2 expression was higher in human OS tissue than in chondroma tissue, while cleaved-caspase 3 expression is relatively lower in OS. Representative images are shown; scale bars, 100 μm. **h** qRT-PCR demonstrated the differential expression of CREB3 at the mRNA level between osteosarcoma and chondroma tissues (n = 10), (*P < 0.01, Student’s *t*-test). **i** Schematic illustration of the complementary sequence between miR-203a-3p and CREB3. Mutated nucleotides of CREB3 3’UTR are shown in lowercase letters. **j** HEK-293 cells were cotransfected with miR-203a-3p mimics (or N.C.) and a luciferase reporter construct containing wild-type (or mutated) CREB3 3′-UTRs. The luciferase activities of the cells were detected. **k** The protein levels of CREB3, c-Jun, Bcl-2, mmp9, mmp2, E-cadherin, N-cadherin and Vimentin in OS cells transfected with miR-203a-3p sponge (or N.C. sponge) or pre-mir-203a-3p (or N.C.) were respectively evaluated by Western blotting. **l** HOS and 143B cells were transfected with sh-circTADA2A or cotransfected with both sh-circTADA2A and the miR-203a-sponge. Western blotting was used to detect the ability of miR-203a-3p to rescue the expression of CREB3, c-Jun, Bcl-2, mmp9, mmp2, E-cadherin, N-cadherin and Vimentin at the protein level. Data are from three independent experiments (mean ± SEM) (**d**-**f and j**) or are representative of three independent experiments with similar results (**g, k and l**) (*P < 0.01 vs control or as indicated by Student’s t-test)

To explore whether circTADA2A enhanced migration, invasion and proliferation in OS by interacting with miR-203a-3p, rescue experiments were performed. Both HOS and 143B cells were stably cotransfected with sh-circTADA2A and a miR-203a-3p sponge (Additional file 2: Figures S3a and S3b), and Transwell migration and Matrigel invasion assays (Fig. 5a) as well as a wound-healing assay (Fig. 5b) were performed. The results revealed that the impairments of migration and invasion were blocked by the exogenous downregulation of miR-203a-3p expression. Accordingly, we found miR-203a-3p sponge could partly reverse the EMT process impaired by sh-circTADA2A, evidenced by the change in mmp2, E-cadherin, N-cadherin and Vimentin protein levels (Fig. 6l and Additional file 4: Figures S6e and S6 g). The zymography assay results revealed that the activity of both mmp2 and mmp9 were decreased by sh-circTADA2A, which could in turn be reversed by the miR-203a-3p sponge (Additional file 2: Figures S3c). In addition, as shown in Fig. 5c-e, we found that the inhibition of miR-203a-3p could partly attenuate the sh-circTADA2A-induced reduction in OS cell viability, as evidenced by the results of the CCK-8 assay, colony formation assay and soft agar assay, which is a three-dimensional environment that imitates the tissue environment where OS grows in the human body. Interestingly, with the increasing duration from 7 days to 14 days, this rescue effect tended to become much more obvious in soft agar assay (Fig. 5e). Moreover, a significant decrease in the apoptotic cell proportion was observed in the sh-circTADA2A and miR-203a-3p sponge cotreatment group compared with the circTADA2A knockdown only group (Fig. 5f and Additional file 2: Figure S3d). These findings suggest that circTADA2A promotes OS progression partly by abolishing the antitumor effects of miR-203a-3p.

### CREB3 is a direct target of miR-203a-3p and is considered an oncogene in OS

According to the theory of competing endogenous RNA (ceRNA), circTADA2A expression should have a positive correlation with the expression of its target genes. To elucidate which target gene was regulated by miR-203a-3p in OS, we screened the transcriptome by RNA-seq, focusing on the differentially expressed genes between HOS cells with stable knockdown of circTADA2A expression and control HOS cells (Fig. 6a and b), which revealed 261 mRNAs with downregulated expression. The corresponding Circos plots, GO analysis and KEGG analysis are shown in Additional file 5: Figures S4a-S4d. Bioinformatic analysis using the TargetScan database revealed 947 potential target genes of miR-203a-3p. Six genes (SFRP1, TYMS, LMNB2, SLC25A23, HMGN2 and CREB3) were selected from the intersection between the RNA-seq-identified downregulated mRNAs and the potential target genes of miR-203a-3p in the TargetScan database (Fig. 6c). To confirm this result, the mRNA expression of the six selected genes was examined in stable HOS and 143B cells (Fig. 6d and e). qRT-PCR results revealed that circTADA2A silencing significantly decreased the mRNA expression of 4 of the genes in the HOS cell line compared with the control, while only 2 of the genes exhibited downregulated expression in the 143B cell line, with HMGN2 and CREB3 included in both clusters. We next designed 6 siRNAs specifically targeting these 6 genes, transfected them separately into HOS and 143B cells, and performed an apoptosis assay after 48 h. As shown in Fig. 6f and Additional file 6: Figure S5a, the downregulation of CREB3 expression enhanced apoptosis, while the knockdown of the other genes failed to enhance apoptosis in both HOS and 143B cell line. To expand this finding, si-CREB3 was transfected into other OS cell lines, including U2OS, SJSA-1 and MG63 cells, and similar results were obtained (Additional file 6: Figure S5b). Although no significant difference was found in the SJSA-1 cell line, an increasing trend in the apoptosis rate after CREB3 knockdown after 48 h was observed.

We then assessed the role of CREB3 in OS cell lines in vivo and in vitro*.* Functionally, lower levels of CREB3 led to the impairment of migration and invasion, as determined by Transwell migration and Matrigel invasion assays along with a wound-healing assay (Additional file 6: Figures S5c and S5d). In addition, colony formation and CCK-8 assays revealed the critical role of CREB3 in promoting proliferation in the OS cells (Additional file 6: Figures S5e and S5f). Meanwhile, we constructed stable 143B cell lines transfected with sh-CREB3 or N.C., and equal amounts of the cells were subcutaneously injected into 4-week-old BALB/c-nu mice. As expected, CREB3 knockdown significantly inhibited tumor growth (Additional file 6: Figure S5 g). Interestingly, we found that the mRNA expression of CREB3 was higher in OS tissue than in chondroma tissue (Fig. 6h), which was further confirmed by immunohistochemistry (Fig. 6g). To investigate whether miR-203a-3p could directly interact with CREB3, we generated 3′-UTR sensors and cotransfected HEK-293 cells with the miR-203a-3p mimics. Reduced luciferase activity by the CREB3 3′-UTR was observed with the overexpression of miR-203a-3p. By comparison, we measured much higher luciferase activity when a mutated form of the CREB3 3′-UTR (disrupted the sequence of the miR-203a-3p binding site) was used (Fig. 6i and j). These lines of evidence suggest that CREB3 is a driver gene in OS and is likely to be the direct target of miR-203a-3p.

### C-Jun is enhanced by CREB3 and regulates the activity of mmp9 and Bcl-2

Previous studies have indicated that CREB3 can bind directly to the c-Jun promoter and subsequently enhance mmp9 activity in cervical cancer cells, which contributes to cervical cancer progression [20]. Nine CREB3 binding sites in the c-Jun promoter with high scores were predicted by the JASPAR database (Additional file 4: Figure S6a). To assess whether CREB3 could interact with c-Jun in OS, we constructed a c-Jun promoter plasmid, which was cotransfected into HOS and 143B cells with si-CREB3 at different concentrations. Surprisingly, we found that si-CREB3 reduced c-Jun promoter activity in a dose-dependent manner, suggesting that CREB3 could regulate the transcriptional activity of c-Jun in OS (Additional file 4: Figure S6b and S6c). It is widely accepted that mmp9 and Bcl-2 can be regulated by c-Jun [20, 35–37]. As expected, the results of immunohistochemistry demonstrated higher abundances of CREB3, c-Jun, mmp9 and Bcl-2 in OS than in chondroma (Fig. 6g). We then used OS cells with stable knockdown or overexpression of miR-203a-3p to evaluate the expression of CREB3, c-Jun, mmp9 and Bcl-2. As shown in Fig. 6k and Additional file 4: Figure S6d and S6f, both the mRNA and protein levels of CREB3, c-Jun, mmp9 and Bcl-2 were negatively correlated with the expression of miR-203a-3p. We further determined that the inhibition of miR-203a-3p could partly reverse the downregulated expression of these genes at the mRNA and protein levels (Fig. 6l and Additional file 4: Figure S6e and S6 g). Together, these results indicate that the CREB3-c-Jun-mmp9/Bcl-2 axis is regulated by circTADA2A through miR-203a-3p.

### CircTADA2A promotes OS progression via CREB3

**Fig. 7:**
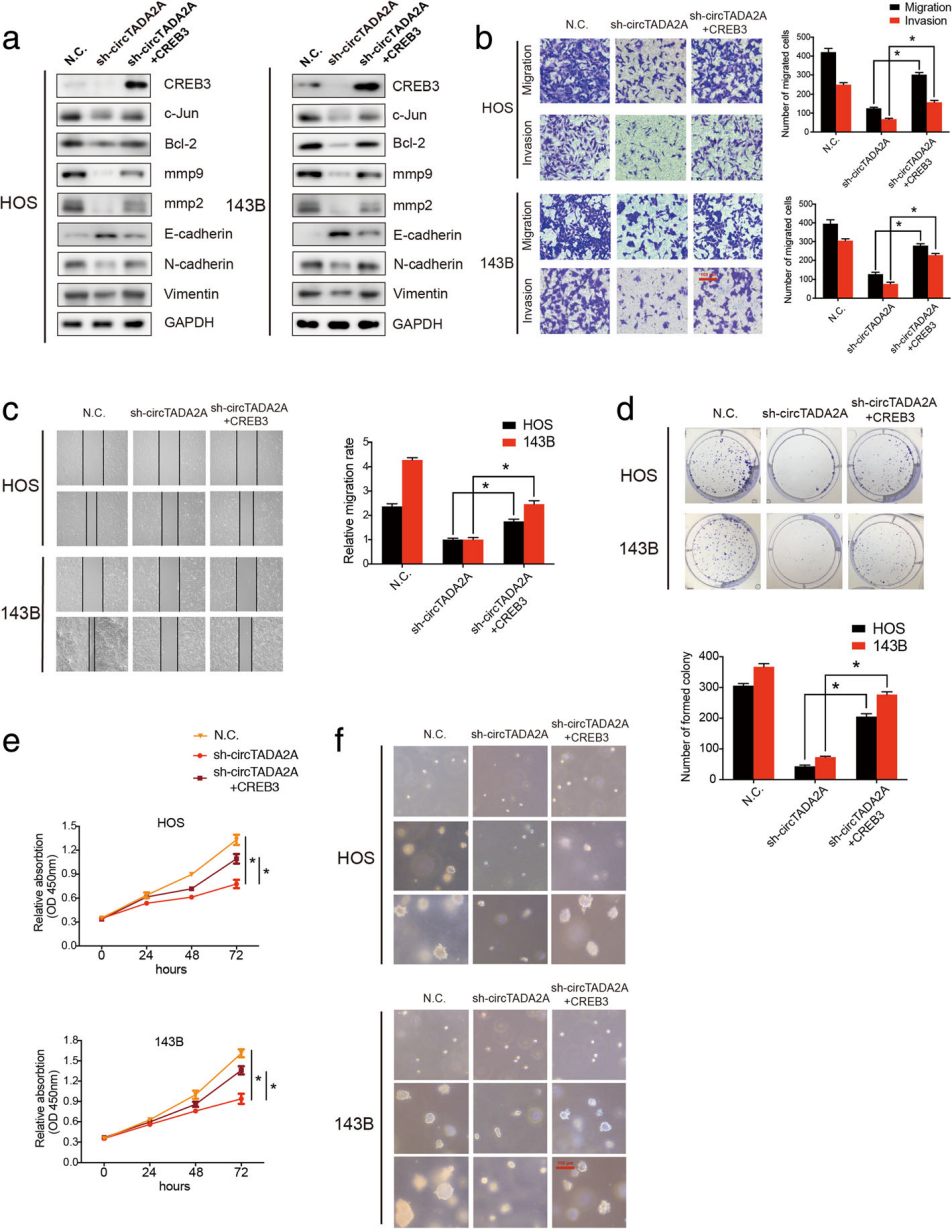
CREB3 functions as a driver gene in OS cells. **a** Western blotting demonstrated the rescue ability of CREB3 on sh-circTADA2A knockdown in HOS and 143B cells. Protein levels of CREB3, c-Jun, Bcl-2, mmp9, mmp2, E-cadherin, N-cadherin and Vimentin were determined. **b** Effects of circTADA2A knockdown on cell migration and invasion were abrogated by CREB3 overexpression. Migration and invasion of OS cells cotransfected with sh-circTADA2A and CREB3 or control shRNA(N.C.) were evaluated by Transwell migration and Matrigel invasion assays, respectively; scale bars, 100 μm. **c** The effects of sh-circTADA2A and CREB3 overexpression on cell migration were determined by a wound-healing assay in OS cells. **d** CREB3 overexpression stimulated the growth of circTADA2A knockdown-mediated cells, as determined by a colony formation assay. **e** CCK-8 assay demonstrated the cell viability alteration of HOS and 143B cells under the effect of circTADA2A silencing and CREB3 overexpression. **f** The proliferation ability of stable OS cells was evaluated by soft agar colony formation assay at 0, 7, and 14 days; scale bars, 100 μm. Data are from three independent experiments (mean ± SEM) (**b-e**) or are representative of three independent experiments with similar results (**a and f**) (*P < 0.01 vs control or as indicated by Student’s t-test)

To further investigate whether circTADA2A facilitates OS progression by targeting CREB3, a CREB3 overexpression plasmid was constructed and transfected into circTADA2A-silenced HOS and 143B cells. qRT-PCR results revealed that CREB3 was able to partly reverse the loss of the expression of c-Jun, mmp9 and Bcl-2 caused by the circTADA2A knockdown (Additional file 7: Figure S7a), and this effect was further confirmed at the protein level via Western blotting (Fig. 7a and Additional file 7: Figure S7b and S7c). We next explored whether CREB3 could improve the phenotypes of the circTADA2A-deficient OS cells. Functionally, migratory and invasive abilities were obviously rescued by CREB3 (Fig. 7b and c). Interestingly, the change in mmp2, E-cadherin, N-cadherin and Vimentin expression also demonstrated the vital role of CREB3 in restoring the impairment of EMT induced by sh-circTADA2A (Fig. 7a and Additional file 7: Figure S7b and c). Next, we performed a zymography assay to evaluate mmp2 and mmp9 activity (Additional file 7: Figure S7d) and found that CREB3 could upregulate the impaired activity of both mmp2 and mmp9 caused by sh-circTADA2A. In addition, we determined that the colony-forming ability of the cells overexpressing CREB3 was significantly enhanced compared with that of the sh-circTADA2A-treated cells (Fig. 7d and f). Additionally, the CCK-8 assay results revealed that OS cell viability could be ameliorated by the expression of CREB3 (Fig. 7e). Altogether, these findings suggest that circTADA2A promotes the migration, invasion and proliferation of OS cells mainly by targeting CREB3.

### CircTADA2A enhances the growth and metastasis of xenograft tumors in vivo

**Fig. 8:**
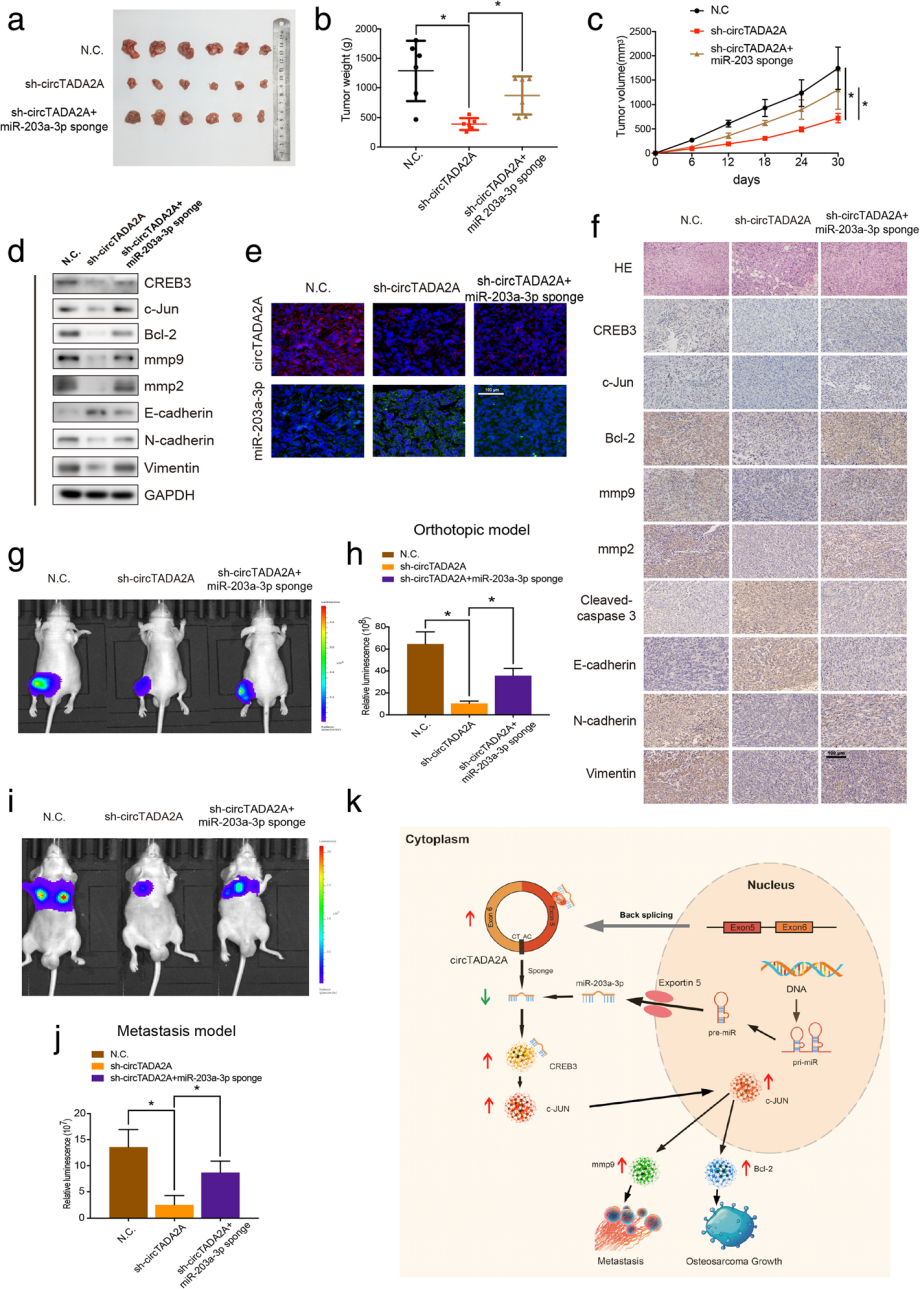
CircTADA2A functions as a miR-203a-3p sponge to promote tumorigenesis in vivo. **a** Nude mice were respectively injected with an equal amount of 5 × 10^6^ stable control cells or cells transfected with circTADA2A shRNA or cotransfected with circTADA2A shRNA and miR-203a-3p sponge subcutaneously. After 30 days, tumors were dissected and photographed. **b** Tumor weight was calculated on the day mice were euthanized. Data represents the mean ± SEM (*n* = 6 each group). **c** Tumor volumes (ab^2^/2) were recorded every six days since the day when mice were injected the stable OS cells. Data represents the mean ± SEM (n = 6 each group). **d** Western blotting demonstrated the protein levels of CREB3, c-Jun, Bcl-2, mmp9, mmp2, E-cadherin, N-cadherin and Vimentin in tumors from different groups. **e** FISH demonstrated the relative expression levels and localization of circTADA2A and miR-203a-3p in the tumors from the mice. Scale bars, 100 μm. **f** H&E staining and immunohistochemistry (IHC) revealed the structure of OS in mice and relative protein levels of CREB3, c-Jun, Bcl-2, mmp9, mmp2, Cleaved-caspase3, E-cadherin, N-cadherin and Vimentin in tumors of different groups. Scale bars, 100 μm. **g & h** 143B cells were labeled with both GFP and luciferase. In vivo bioluminescence imaging system showed the orthotopic xenograft tumor in 3 groups of mice injected with 3 different stable 143B cells. Representative images and a histogram are shown (*n* = 9 each group). **i & j** Lung metastasis of mice injected with different stable 143B cells via the tail vein was detected using an in vivo bioluminescence imaging system. Representative images and a histogram are shown (n = 9 each group). **k** Schematic illustration of the circTADA2A/miR-203a-3p/CREB3 axis. Data are from three independent experiments (mean ± SEM) (**h and j**) or are representative of three independent experiments with similar results (**d**-**f**, **g and i**) (*P < 0.01 vs control or as indicated by Student’s t-test)

To investigate the functions of circTADA2A and miR-203a-3p in vivo, a xenograft tumor model was established. We used stable 143B cells transfected with N.C. or sh-circTADA2A or cotransfected with sh-circTADA2A and the miR-203a-3p sponge. FISH confirmed the knockdown of circTADA2A expression and the suppression of miR-203a-3p (Fig. 8e). The tumors derived from the circTADA2A-deficient cells were much smaller in size and weighed less than the control tumors. Surprisingly, the miR-203a-3p sponge significantly reversed the impairments in tumor size and weight caused by circTADA2A silencing (Fig. 8a-c). Total RNA and protein were extracted from the tumors, and qRT-PCR and Western blotting respectively provided powerful evidence showing changes in the mRNA and protein expression levels of CREB3, c-Jun, mmp9 and Bcl-2 (Fig. 8d and Additional file 8: Figure S8a and S8b). CircTADA2A silencing predominantly reduced both CREB3, c-Jun, Bcl-2 and mmp9 expression at the mRNA and protein level, which could be reversed by the miR-203a-3p sponge. Accordingly, the mean immunopositive area for CREB3, c-Jun, mmp9, Bcl-2 and cleaved-caspase 3 was decreased under the influence of sh-circTADA2A, as determined by immunohistochemistry, and the inhibition of miR-203a-3p again counteracted this change (Fig. 8f). We next performed TUNEL assays to evaluate the cell viability of the tumor cells and found that, as shown in Additional file 8: Figure S8c, compared with the control tumors, the circTADA2A knockdown tumors revealed an increase apoptosis that could be partly attenuated by miR-203a-3p sponge. Considering that OS generally occurs and develops in the femur or tibia, we further constructed an orthotopic xenograft tumor model by injecting 143B cells labeled with both a luminescent dye and GFP into the cavity of the tibia. In vivo bioluminescence imaging demonstrated that the knockdown of circTADA2A expression attenuated the proliferation of OS cells in situ, while miR-203a-3p suppression alleviated this impairment (Fig. 8g and h), which is similar to the results of the subcutaneous model described in Fig. 8a-c.

To further investigate the metastatic potential of circTADA2A in vivo, 143B cells (labeled with both a luminescent dye and GFP) with or without stable circTADA2A knockdown or with both circTADA2A and miR-203a-3p silenced were generated and injected into nude mice via the tail vein. By detecting the GFP signal, flow cytometry confirmed the presence of CTCs in the circulation of nude mice (Additional file 8: Figure S8d), with CTCs or CTC cluster further shown using fluorescence microscope (Additional file 8: Figure S8e). Moreover, pervasive lung metastasis was detected in the control group compared with the sh-circTADA2A group 4 weeks after injection, as determined by in vivo bioluminescence imaging (Fig. 8i and j). Interestingly, the downregulation of miR-203a-3p expression dramatically reversed the metastatic tumor size. Additionally, we found that the mesenchymal phenotype was attenuated by circTADA2A knockdown and enhanced by the miR-203a-3p sponge; changes in the expression of mmp2, E-cadherin, N-cadherin and Vimentin, as determined by Western blotting and IHC, were also found (Fig. 8d-f and Additional file 8: Figure S8b). These data confirm that circTADA2A may play a critical role in promoting the proliferation and metastasis of OS in vivo.

## Discussion

CircRNA is a type of noncoding RNA that has been identified in the past several years. Recently, an increasing number of studies have revealed the important roles of circRNA in different biological processes, especially carcinogenesis, and in the progression and metastasis of cancers such as lung adenocarcinoma [11], bladder cancer [30] and gastric cancer [38]. A previous study reported that a large number of circRNAs are differentially expressed between OS cell lines and the corresponding noncancerous cell line [26]. However, the functions of these circRNAs remain largely unknown. In this study, we focused on the role and underlying mechanism of circTADA2A, which is dramatically upregulated in OS cells and tissue.

The TADA2A gene encodes a transcriptional activator adaptor, a crucial component of the general control nonderepressible 5 (GCN5) histone acetyltransferase complex [39]. Previous studies have reported that TADA2A plays an important role in chromatin remodeling and contributes to the reduction in TP53 transcriptional activity. Additionally, TADA2A promotes apoptosis in response to DNA damage via XRCC6 acetylation [40]. However, no studies have revealed the role of circTADA2A in any disease or biological process. It is widely accepted that circRNAs are derived from several exons of specific genes [6, 10]. Here, we determined that circTADA2A, which is abundantly expressed in OS, originated from exons 5 and 6 of TADA2A and formed a ring structure by connecting the 3′ and 5′ splice sites. Even under treatment with RNase R, circTADA2A could still be detected with little degradation, demonstrating the stability of circRNAs reported in previous studies [41]. The expression of circTADA2A has been determined to be generally upregulated in OS compared with chondroma, indicating that circTADA2A might be associated with the development of OS.

CircRNAs are essential members of ceRNA networks and have been suggested to have multiple functions, among which the ‘circRNA-miRNA-mRNA’ axis, also known as the ‘miRNA sponge’, is the most frequently reported [28, 30, 38, 42]. This type of regulation is highly complex because of the variability in the interactions between circRNAs and miRNAs. The most well-known circRNA is CDR1a, which is studied in the neuroscience field; CDR1a contains 63 binding sites for miR-7 and is considered one of the most powerful antagonists with miRNA-binding capacity [43]. In our study, 20 potential miRNAs with several binding sites for circTADA2A were identified via a computational algorithm. RIP and luciferase assays have been shown efficiently identify the precise and authentic interactions between circRNAs and miRNAs [27]. We found that miR-203a-3p not only had a high binding capacity with circTADA2A in both HOS and 143B cells but also demonstrated critical functions in OS progression and metastasis. Furthermore, our findings suggest that circTADA2A influences the biological behavior of OS by targeting CREB3 via miR-203a-3p. CREB3 was first identified as a protein that plays a vital role in the process of HSV infection [19]. In addition, CREB3 is reported to be involved in the malignant phenotype of prostate cancer via the androgen receptor [21]. In addition, CREB3 can accelerate breast cancer metastasis by enhancing the transcriptional activation of ER-Golgi trafficking genes [44]. In this work, our study is the first to verify CREB3 as a driver gene in OS through loss-of-function experiments in vivo *and* in vitro. We demonstrated that CREB3 could bind to the promoter of c-Jun and subsequently enhance the expression of mmp9, an important biomarker for tumor metastasis, and Bcl-2, a pivotal antiapoptotic molecule in the plasma. Therefore, by enhancing the transcriptional activity of CREB3 and sponging miR-203a-3p, circTADA2A promotes both the progression and metastasis of OS.

Some noncoding RNAs can be stably and repeatedly detected in body fluids such as plasma, which makes it possible for noncoding RNAs to be essential biomarkers or even therapeutic targets in certain diseases [45–47]. Therefore, whether circTADA2A can be stably examined in body fluids and whether circTADA2A expression has a positive correlation with the clinical stage of OS need further investigation, and we will continue to focus on these issues. Although the oncogenic effects of circTADA2A in OS were demonstrated in this research, we cannot exclude the possibility that there might be other critical circRNAs with upregulated expression involved in the initiation and development of OS.

## Conclusion

In summary, our data reveal that circTADA2A expression is strongly increased in OS tissues and cell lines. Functionally and mechanistically, circTADA2A promotes the progression and metastasis of OS by sponging miR-203a-3p and targeting CREB3, which has been identified by us to be an oncogene in OS. Moreover, an in vivo intervention targeting the circTADA2A-miR-203a-3p-CREB3 axis demonstrated potency as an OS-targeted therapy. Our findings are the first to elucidate the role of circTADA2A in OS, which may offer a novel therapeutic target for OS treatment.

## Additional files

Additional file 1: **Figure S1.** The effects of knocking down several microRNAs on the apoptosis of OS cells. **a** HOS and 143B cells were transfected with various microRNA mimics or mimics N.C. After 48 h, apoptotic cells were detected by flow cytometry with Annexin V-FITC/PI staining. Histograms are shown in Fig. 3. **b** Schematic illustration shows the complementary sequence between miR-203a-3p and circTADA2A. CircTADA2A Mut1, Mut2 and Co-Mut sequences are shown in lowercase letters. Data are representative of three independent experiments with similar results (**a**). (TIF 2763 kb)

Additional file 2: **Figure S3.** The rescue effect of miR-203a-3p on circTADA2A on OS cells. **a & b** HOS and 143B cells were stably transfected with sh-circTADA2A or cotransfected with sh-circTADA2A and miR-203a-3p sponge. circTADA2A and miR-203a-3p expression was detected by qRT-PCR. **c** Both mmp2 and mmp9 activity of stably transfected OS cells were exhibited in zymography assay. **d** The effects of circTADA2A knockdown and miR-203a-3p sponge rescue on circTADA2A silencing were evaluated by apoptosis assay. Apoptotic rates are shown in Fig. 5f. Data are from three independent experiments (mean ± SEM) (**a and b**) or are representative of three independent experiments with similar results (**c and d**) (*P < 0.01 vs control or as indicated by Student’s t-test). (TIF 2156 kb)

Additional file 3: **Figure S2.** The transfection effects of miR-203a-3p. **a & b** The miR-203a-3p alteration of both HOS and 143B cells stably transfected with N.C. sponge or miR-203a-3p sponge or N.C. or pre-miR-203a-3p was determined by qRT-PCR. Histograms show the fold-change in miR-203a-3p expression. **c** Zymography assay demonstrated the activity of mmp2 and mmp9 in both stable HOS and 143B cells. Data are from three independent experiments (mean ± SEM) (**a and b**) or are representative of three independent experiments with similar results (**c**) (*P < 0.01 vs control or as indicated by Student’s t-test). (TIF 986 kb)

Additional file 4: **Figure S6.** C-Jun is regulated by CREB3. **a** Effective binding sites (Relative score > 0.7) were predicted using the JASPAR database. **b & c** OS cells were cotransfected with the c-Jun reporter gene plasmid and the indicated amount of si-CREB3, followed by the evaluation of luciferase assay after 24 h. **d** The mRNA levels of CREB3, c-Jun, mmp9 and Bcl-2 of OS cells transfected miR-203a-3p sponge (or N.C. sponge) or pre-mir-203a-3p (or N.C.) were respectively evaluated by qRT-PCR. **e** HOS and 143B cells were transfected with sh-circTADA2A or cotransfected with both sh-circTADA2A and miR-203a-sponge. qRT-PCR was used to detect the rescue ability of miR-203a-3p on the expression of CREB3, c-Jun, mmp9 and Bcl-2 in mRNA level. **f & g** Gray analysis of Western blotting results is shown in Fig. 6. Data are from three independent experiments (mean ± SEM) (**b-g**) (*P < 0.01 vs control or as indicated by Student’s t-test). (TIF 2397 kb)

Additional file 5: **Figure S4.** Bioinformatic analysis of RNA sequencing. **a** Circos plots show the expressed genes in chromosomes. Outer: chromosomes; inner: plus strand (red) and minus strand (green). **b** Go analysis of differentially expressed genes were shown. **c & d** KEGG analysis of differentially expressed genes. (TIF 2356 kb)

Additional file 6: **Figure S5.** CREB3 functions as a driver gene in Osteosarcoma. **a** HOS and 143B cells were transfected with si-SFRP1 (or si-TYMS or si-LMNB2 or si-SLC25A23 or si-HMGN2 or si-CREB3 or N.C.). After 48 h, the apoptosis rate of OS cells with knockdown of certain genes was determined by apoptosis assay with Annexin V-FITC/PI staining. Histograms are shown in Fig. 6f. **b** OS cells including U2OS, SJSA-1 and MG63 were transfected with si-CREB3, and then an apoptosis assay was performed with Annexin V-FITC/PI after 48 h. **c** Cells were transfected with si-CREB3, followed by evaluation of the migration and invasion abilities by Transwell migration and Matrigel invasion assays. **d** Wound-healing assay demonstrated the alteration of cell migration rates with the silence of CREB3. **e** Colony formation assay demonstrated the capacity of proliferation in OS cells. **f** Cell viability of OS cells under the effect of si-CREB3 after 24 h, 48 h and 72 h were examined by CCK-8 assay. **g** BALB/c-nu (*n* = 6) mice were respectively subcutaneously injected with 143B cells stably transfected with N.C. and sh-CREB3. Representative images of tumors after 30 days are shown. Data are from three independent experiments (mean ± SEM) (**b-f)** or are representative of three independent experiments with similar results (**a**) (*P < 0.01 vs control or as indicated Student’s t-test). (TIF 6952 kb)

Additional file 7: **Figure S7.** The mRNA, protein levels of some genes and MMP activity in different stable OS cells. **a** Sh-circTADA2A with or without CREB3 overexpression was transfected into OS cells, followed by qRT-PCR detection on CREB3, c-Jun, mmp9 and Bcl-2. The histograms show the relative alteration in mRNA levels. **b & c** Cells were stably transfected with sh-circTADA2A and N.C. (or CREB3). The relative protein levels of CREB3, c-Jun, Bcl-2, mmp9, mmp2, E-cadherin, N-cadherin and Vimentin were analyzed and bands are shown in Fig. 7. **d** Mmp2 and mmp9 activity in different stable OS cells was evaluated by a zymography assay. Data are from three independent experiments (mean ± SEM) (**a-c**) or are representative of three independent experiments with similar results (**d**) (*P < 0.01 vs control or as indicated by Student’s t-test). (TIF 1683 kb)

Additional file 8: **Figure S8.** The role of circTADA2A and miR-203a-3p in osteosarcoma in vivo. **a** The expression of CREB3, c-Jun, mmp9 and Bcl-2 mRNA levels were detected by qRT-PCR. **b** Gray analysis of the protein bands in Western blotting was demonstrated. Proteins were extracted from corresponding tumors. **c** TUNEL assay showed tumor cell death; scale bars, 100 μm. **d** Blood containing CTCs labeled with GFP was collected, lysed by red blood cell lysis buffer, and CTCs were detected by flow cytometry. The histogram shows the percentage of relative GFP-positive cells. **e** Representative images of CTCs (yellow arrows) or CTC clusters (white arrows) in blood lysed by red blood cell lysis buffer. Data are from three independent experiments (mean ± SEM) (**a, b and d**) or are representative of three independent experiments with similar results (**c and e**) (*P < 0.01 vs control or as indicated by Student’s t-test). (TIF 3188 kb)

## Abbreviations

ceRNA: Competing endogenous RNA
circRNA: Circular RNA
CREB3: Cyclic AMP-responsive element-binding protein 3
FISH: Fluorescence in situ hybridization
miRNA: MicroRNA
mmp2: Matrix metallopeptidase 2
mmp9: Matrix metallopeptidase 9
OS: Osteosarcoma
qRT-PCR: Quantitative real-time polymerase chain reaction
sh-RNA: Small hairpin RNA
siRNA: Small interfering RNA
TADA2A: Transcriptional adaptor 2A
