# Supplementary figures and images for: Circular RNA circTADA2A promotes osteosarcoma progression and metastasis by sponging miR-203a-3p and regulating CREB3 expression
Source: Mol Cancer. 2019 Apr 2;18:73. doi: 10.1186/s12943-019-1007-1 (PMC6444890; doi:10.1186/s12943-019-1007-1)

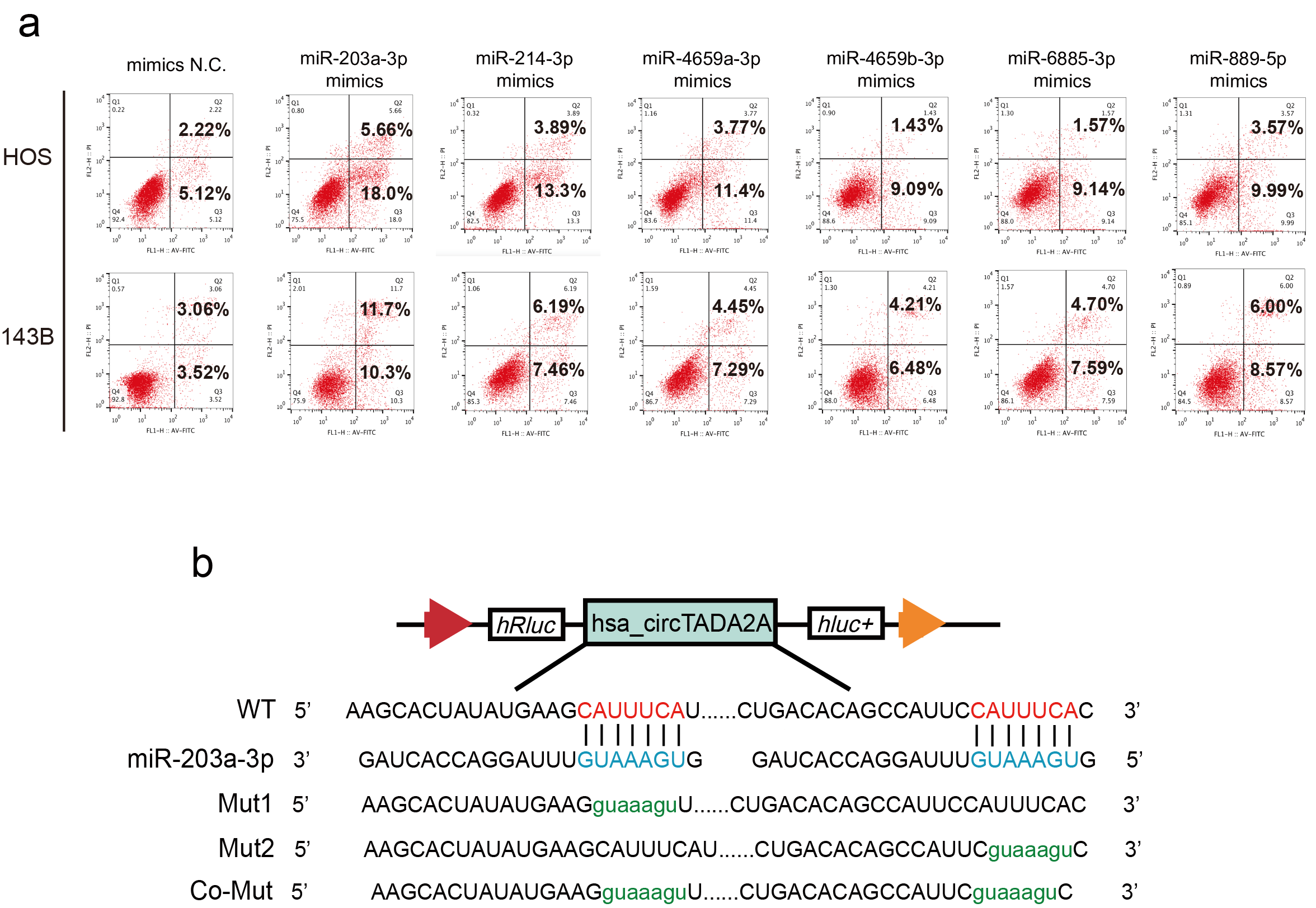

Supplement: Supplementary file 1 — Figure S1. The effects of knocking down several microRNAs on the apoptosis of OS cells. a HOS and 143B cells were transfected with various microRNA mimics or mimics N.C. After 48 h, apoptotic cells were detected by flow cytometry with Annexin V-FITC/PI staining. Histograms are shown in Fig. 3. b Schematic illustration shows the complementary sequence between miR-203a-3p and circTADA2A. CircTADA2A Mut1, Mut2 and Co-Mut sequences are shown in lowercase letters. Data are representative of three independent experiments with similar results (a). (TIF 2763 kb) [file 12943_2019_1007_MOESM1_ESM.tif]

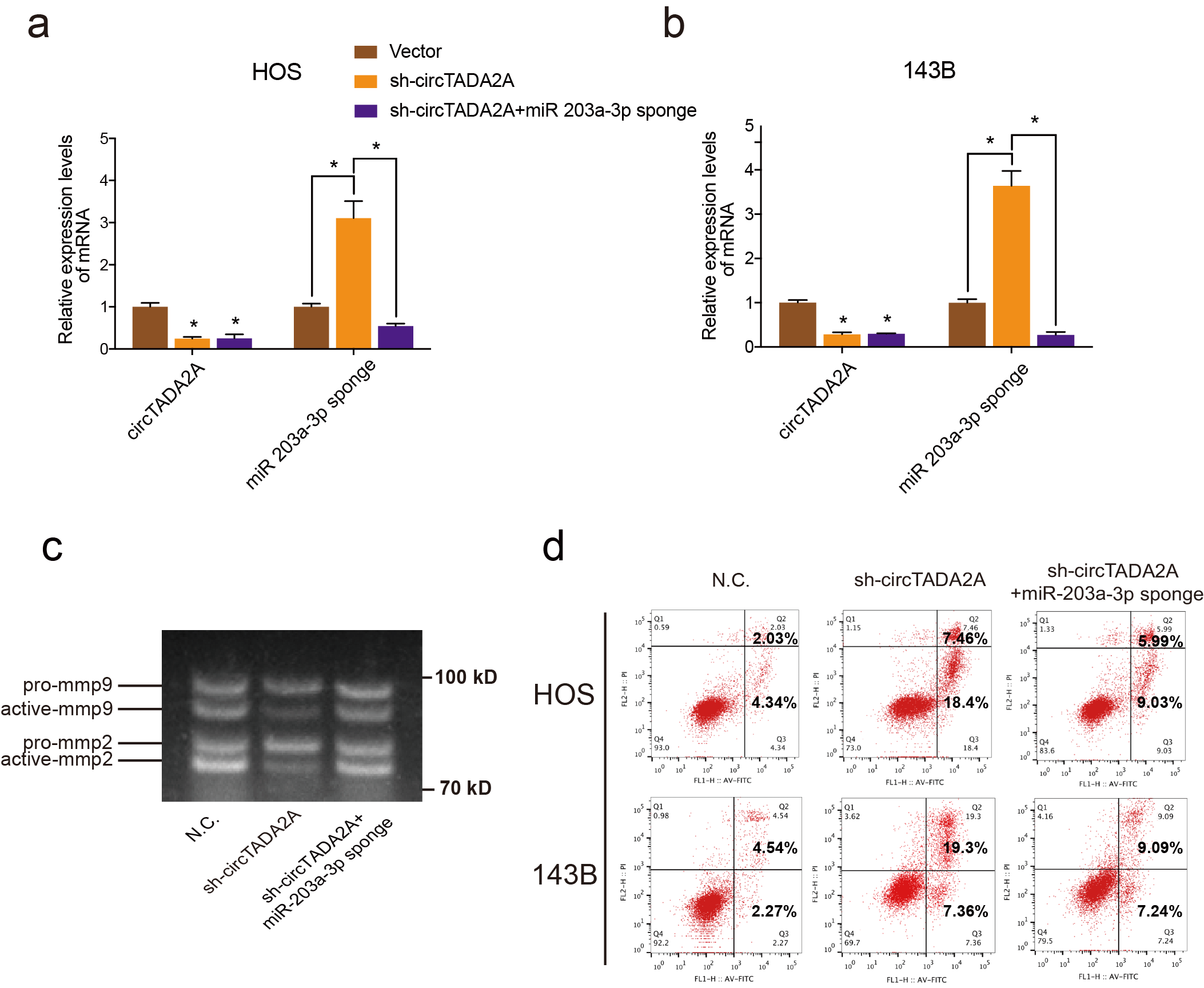

Supplement: Supplementary file 2 — Figure S3. The rescue effect of miR-203a-3p on circTADA2A on OS cells. a & b HOS and 143B cells were stably transfected with sh-circTADA2A or cotransfected with sh-circTADA2A and miR-203a-3p sponge. circTADA2A and miR-203a-3p expression was detected by qRT-PCR. c Both mmp2 and mmp9 activity of stably transfected OS cells were exhibited in zymography assay. d The effects of circTADA2A knockdown and miR-203a-3p sponge rescue on circTADA2A silencing were evaluated by apoptosis assay. Apoptotic rates are shown in Fig. 5f. Data are from three independent experiments (mean ± SEM) (a and b) or are representative of three independent experiments with similar results (c and d) (*P < 0.01 vs control or as indicated by Student’s t-test). (TIF 2156 kb) [file 12943_2019_1007_MOESM2_ESM.tif]

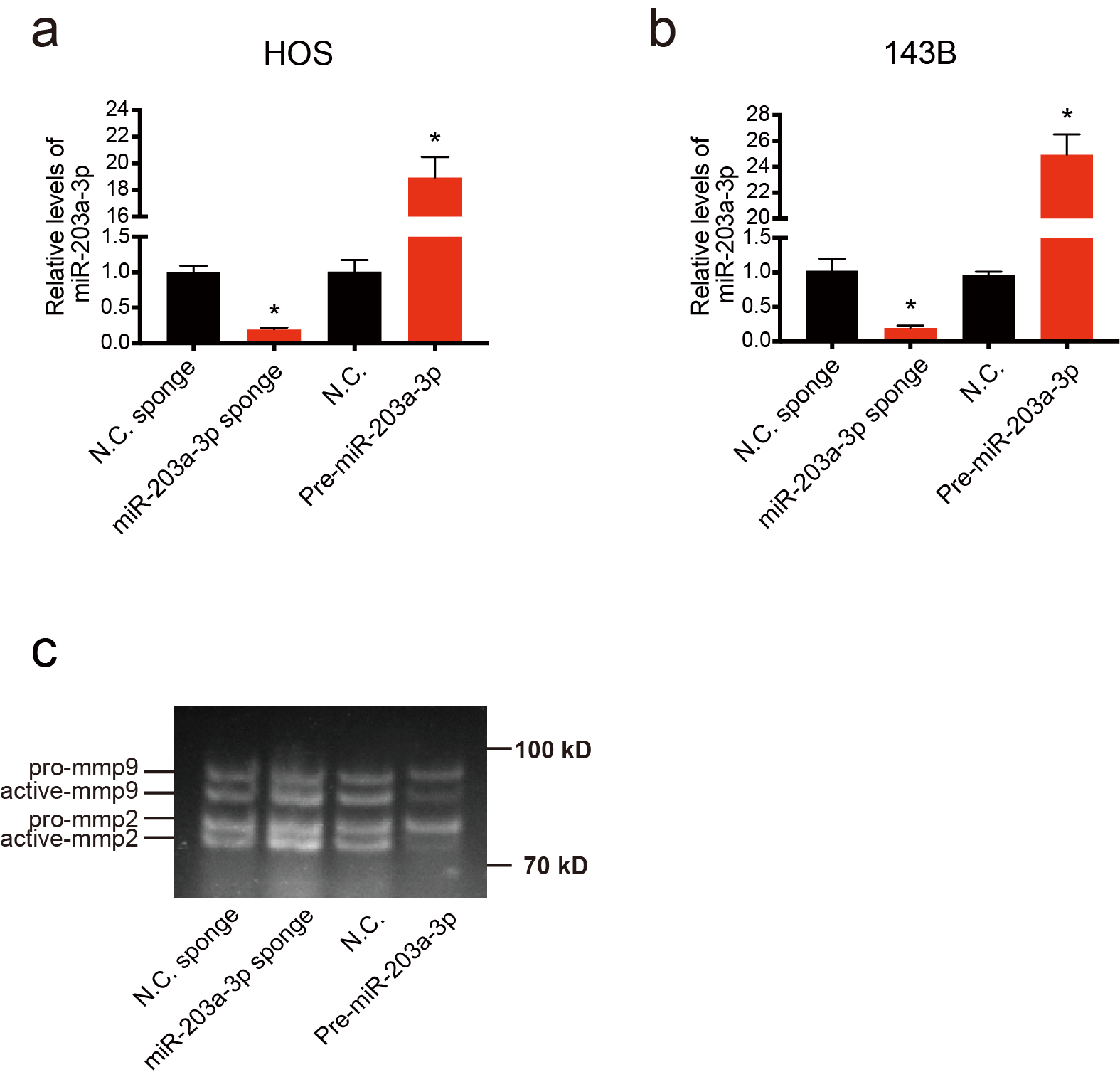

Supplement: Supplementary file 3 — Figure S2. The transfection effects of miR-203a-3p. a & b The miR-203a-3p alteration of both HOS and 143B cells stably transfected with N.C. sponge or miR-203a-3p sponge or N.C. or pre-miR-203a-3p was determined by qRT-PCR. Histograms show the fold-change in miR-203a-3p expression. c Zymography assay demonstrated the activity of mmp2 and mmp9 in both stable HOS and 143B cells. Data are from three independent experiments (mean ± SEM) (a and b) or are representative of three independent experiments with similar results (c) (*P < 0.01 vs control or as indicated by Student’s t-test). (TIF 986 kb) [file 12943_2019_1007_MOESM3_ESM.tif]

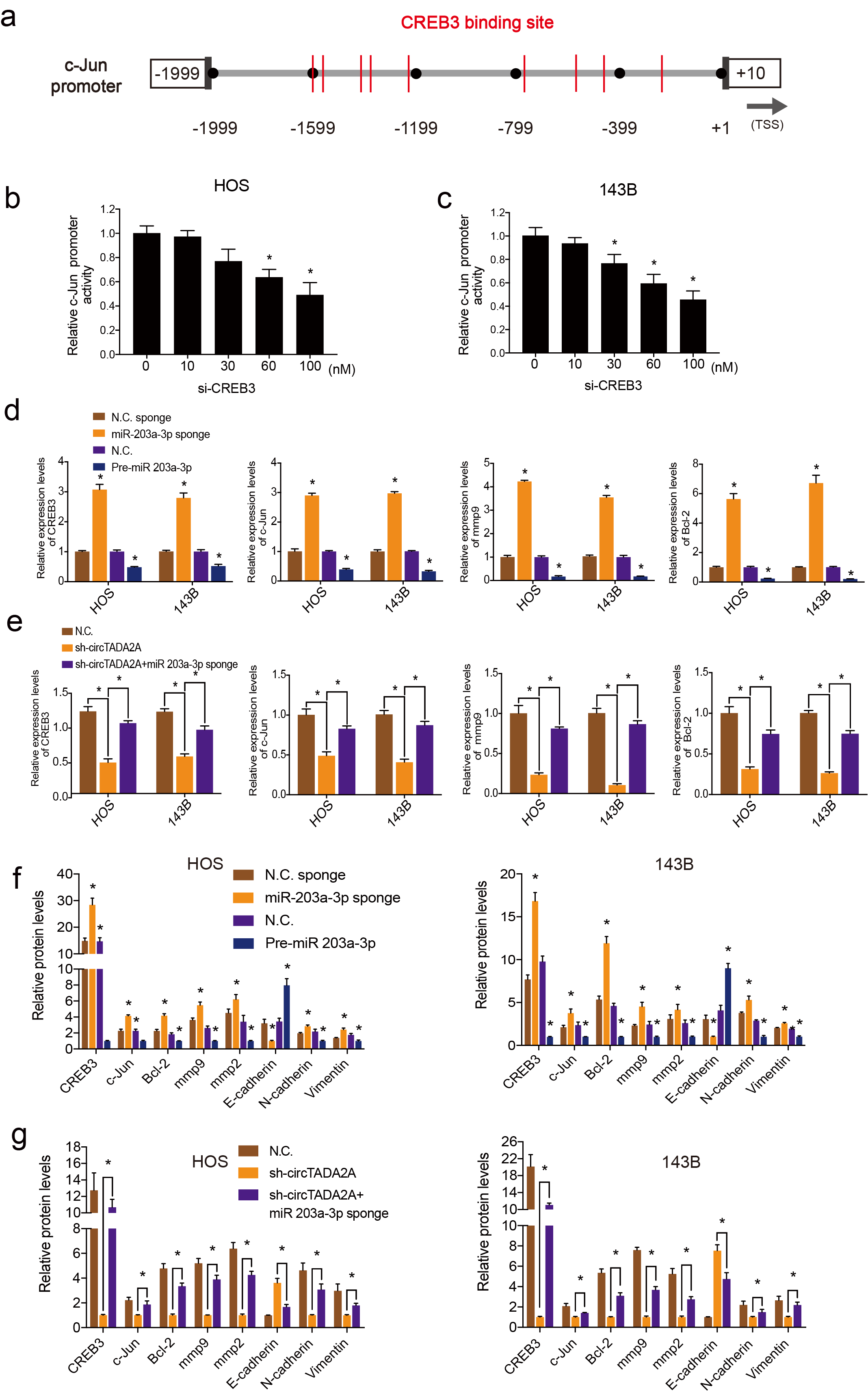

Supplement: Supplementary file 4 — Figure S6. C-Jun is regulated by CREB3. a Effective binding sites (Relative score > 0.7) were predicted using the JASPAR database. b & c OS cells were cotransfected with the c-Jun reporter gene plasmid and the indicated amount of si-CREB3, followed by the evaluation of luciferase assay after 24 h. d The mRNA levels of CREB3, c-Jun, mmp9 and Bcl-2 of OS cells transfected miR-203a-3p sponge (or N.C. sponge) or pre-mir-203a-3p (or N.C.) were respectively evaluated by qRT-PCR. e HOS and 143B cells were transfected with sh-circTADA2A or cotransfected with both sh-circTADA2A and miR-203a-sponge. qRT-PCR was used to detect the rescue ability of miR-203a-3p on the expression of CREB3, c-Jun, mmp9 and Bcl-2 in mRNA level. f & g Gray analysis of Western blotting results is shown in Fig. 6. Data are from three independent experiments (mean ± SEM) (b-g) (*P < 0.01 vs control or as indicated by Student’s t-test). (TIF 2397 kb) [file 12943_2019_1007_MOESM4_ESM.tif]

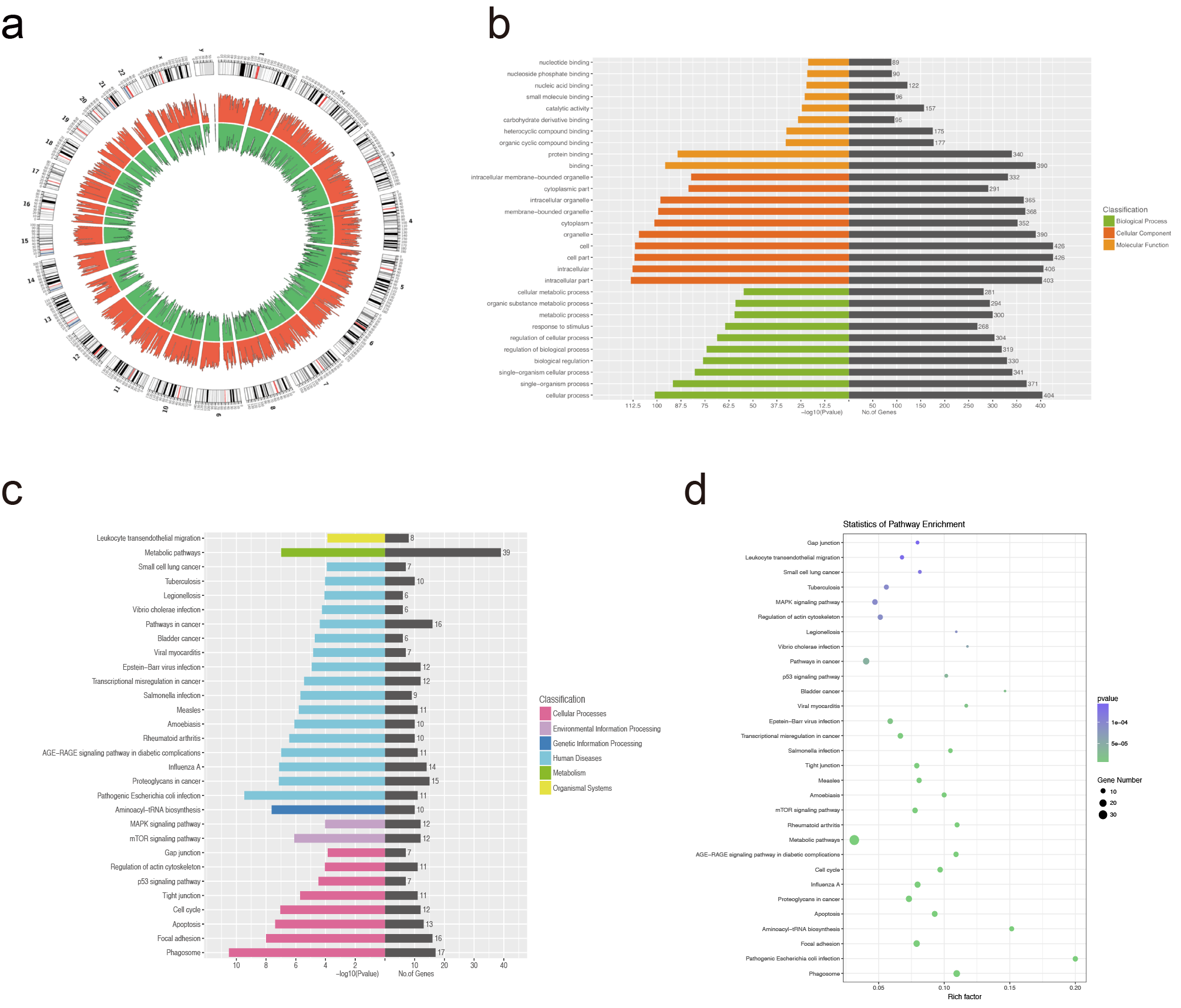

Supplement: Supplementary file 5 — Figure S4. Bioinformatic analysis of RNA sequencing. a Circos plots show the expressed genes in chromosomes. Outer: chromosomes; inner: plus strand (red) and minus strand (green). b Go analysis of differentially expressed genes were shown. c & d KEGG analysis of differentially expressed genes. (TIF 2356 kb) [file 12943_2019_1007_MOESM5_ESM.tif]

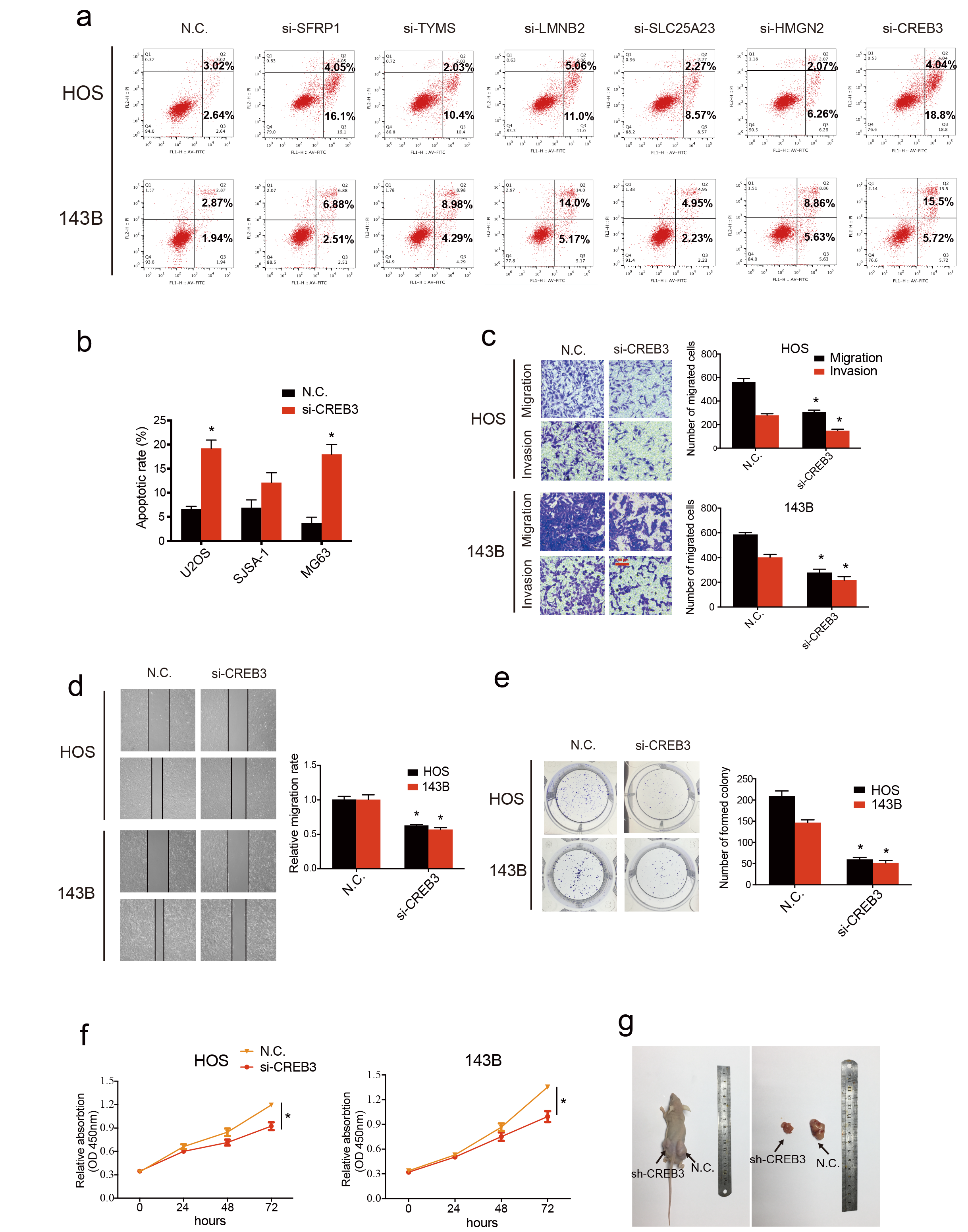

Supplement: Supplementary file 6 — Figure S5. CREB3 functions as a driver gene in Osteosarcoma. a HOS and 143B cells were transfected with si-SFRP1 (or si-TYMS or si-LMNB2 or si-SLC25A23 or si-HMGN2 or si-CREB3 or N.C.). After 48 h, the apoptosis rate of OS cells with knockdown of certain genes was determined by apoptosis assay with Annexin V-FITC/PI staining. Histograms are shown in Fig. 6f. b OS cells including U2OS, SJSA-1 and MG63 were transfected with si-CREB3, and then an apoptosis assay was performed with Annexin V-FITC/PI after 48 h. c Cells were transfected with si-CREB3, followed by evaluation of the migration and invasion abilities by Transwell migration and Matrigel invasion assays. d Wound-healing assay demonstrated the alteration of cell migration rates with the silence of CREB3. e Colony formation assay demonstrated the capacity of proliferation in OS cells. f Cell viability of OS cells under the effect of si-CREB3 after 24 h, 48 h and 72 h were examined by CCK-8 assay. g BALB/c-nu (n = 6) mice were respectively subcutaneously injected with 143B cells stably transfected with N.C. and sh-CREB3. Representative images of tumors after 30 days are shown. Data are from three independent experiments (mean ± SEM) (b-f) or are representative of three independent experiments with similar results (a) (*P < 0.01 vs control or as indicated Student’s t-test). (TIF 6952 kb) [file 12943_2019_1007_MOESM6_ESM.tif]

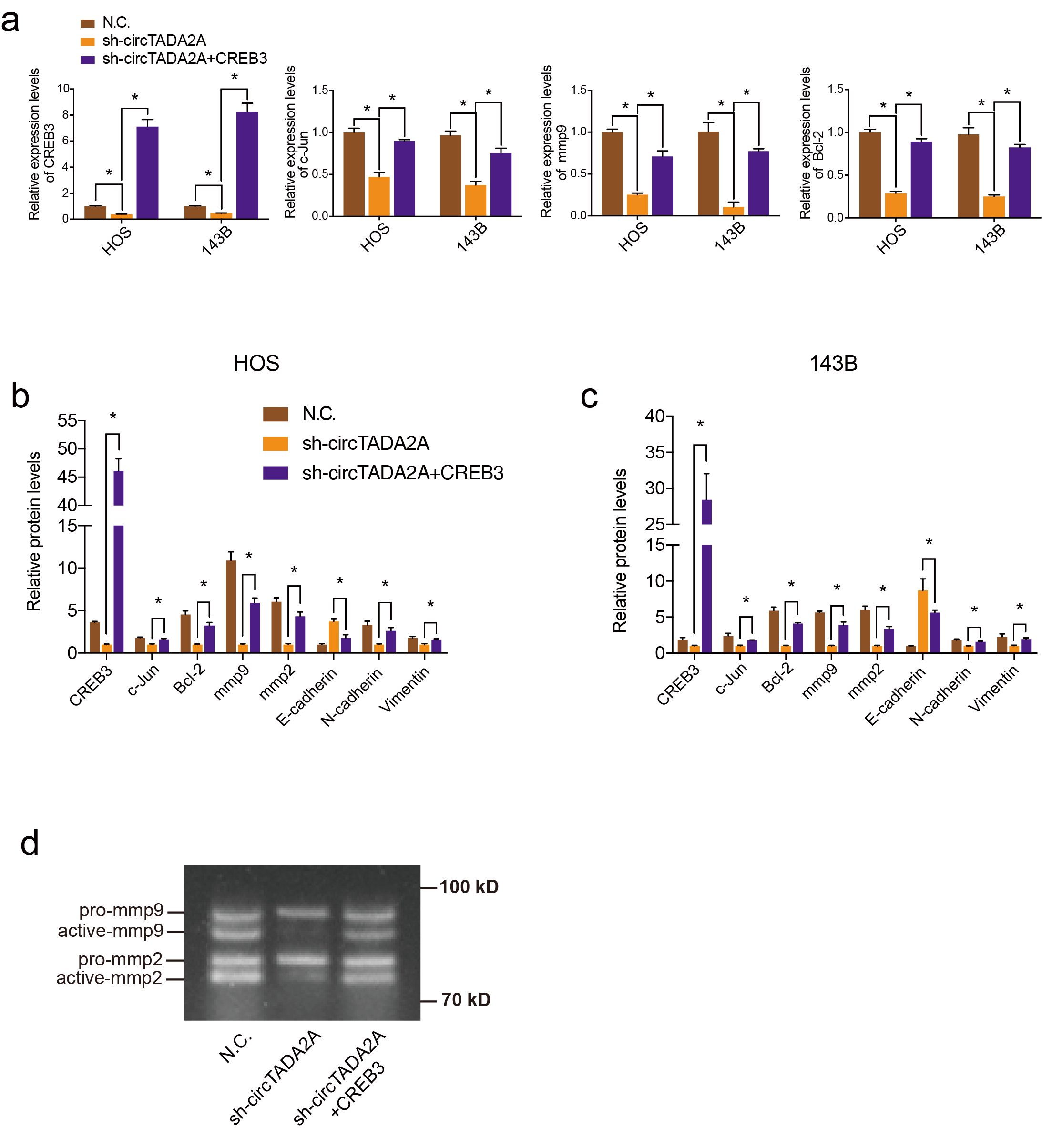

Supplement: Supplementary file 7 — Figure S7. The mRNA, protein levels of some genes and MMP activity in different stable OS cells. a Sh-circTADA2A with or without CREB3 overexpression was transfected into OS cells, followed by qRT-PCR detection on CREB3, c-Jun, mmp9 and Bcl-2. The histograms show the relative alteration in mRNA levels. b & c Cells were stably transfected with sh-circTADA2A and N.C. (or CREB3). The relative protein levels of CREB3, c-Jun, Bcl-2, mmp9, mmp2, E-cadherin, N-cadherin and Vimentin were analyzed and bands are shown in Fig. 7. d Mmp2 and mmp9 activity in different stable OS cells was evaluated by a zymography assay. Data are from three independent experiments (mean ± SEM) (a-c) or are representative of three independent experiments with similar results (d) (*P < 0.01 vs control or as indicated by Student’s t-test). (TIF 1683 kb) [file 12943_2019_1007_MOESM7_ESM.tif]

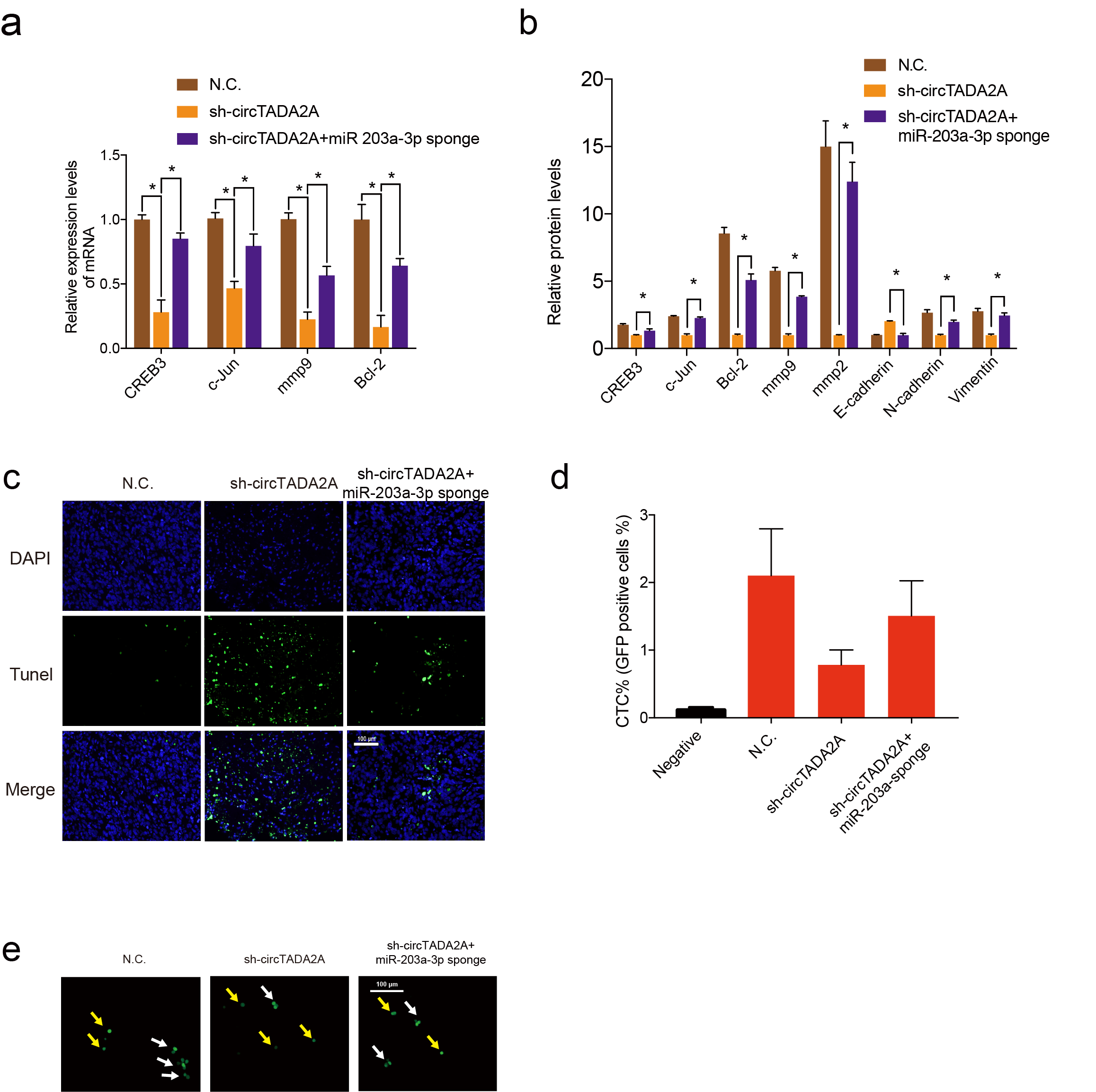

Supplement: Supplementary file 8 — Figure S8. The role of circTADA2A and miR-203a-3p in osteosarcoma in vivo. a The expression of CREB3, c-Jun, mmp9 and Bcl-2 mRNA levels were detected by qRT-PCR. b Gray analysis of the protein bands in Western blotting was demonstrated. Proteins were extracted from corresponding tumors. c TUNEL assay showed tumor cell death; scale bars, 100 μm. d Blood containing CTCs labeled with GFP was collected, lysed by red blood cell lysis buffer, and CTCs were detected by flow cytometry. The histogram shows the percentage of relative GFP-positive cells. e Representative images of CTCs (yellow arrows) or CTC clusters (white arrows) in blood lysed by red blood cell lysis buffer. Data are from three independent experiments (mean ± SEM) (a, b and d) or are representative of three independent experiments with similar results (c and e) (*P < 0.01 vs control or as indicated by Student’s t-test). (TIF 3188 kb) [file 12943_2019_1007_MOESM8_ESM.tif]
